# PRKDC promotes hepatitis B virus transcription through enhancing the binding of RNA Pol II to cccDNA

**DOI:** 10.1038/s41419-022-04852-3

**Published:** 2022-04-25

**Authors:** Yao Fan, Yi Liang, Yu Liu, Hui Fan

**Affiliations:** 1 The Key Laboratory of Molecular Biology of Infectious Diseases designated by the Chinese Ministry of Education, Chongqing Medical University, Chongqing, 400016 China; 2 The Affiliated Traditional Chinese Medicine Hospital of Southwest Medical University, Luzhou, 646000 China

**Keywords:** Infectious diseases, Epigenetics

## Abstract

Hepatitis B virus infection remains a major health problem worldwide due to its high risk of liver failure and hepatocellular carcinoma. Covalently closed circular DNA (cccDNA), which is present as an individual minichromosome, serves as the template for transcription of all viral RNAs and pla ays critical role in viral persistence. Therefore, there is an urgent need to gain broader insight into the transcription regulation of cccDNA. Here, we combined a modified Clustered Regularly Interspaced Short Palindromic Repeats (CRISPR) with an engineered ascorbate peroxidase 2 (APEX2) to identify cccDNA associated proteins systematically in living cells. By functional screening, we verified that protein kinase, DNA-activated, catalytic subunit (PRKDC) was an effective activator of HBV cccDNA transcription in HBV-infected HepG2-NTCP cells and primary human hepatocytes. Mechanismly, PRKDC interacted with POLR2A and POLR2B, the two largest subunits of RNA polymerase II (Pol II) and recruited Pol II to HBV cccDNA minichromosome in a kinase-dependent manner. PRKDC knockdown or inhibitor treatment significantly decreased the enrichment of POLR2A and POLR2B on cccDNA, as well as reducing the levels of cccDNA associated Pol II Ser5 and Ser2 phosphorylation, which eventually inhibited the HBV cccDNA activity. Collectively, these findings give us new insights into cccDNA transcription regulation, thus providing new potential targets for HBV treatment in patients.

## Introduction

Hepatitis B virus (HBV) consistently remains a global health burden due to the risk of developing cirrhosis and hepatocellular carcinoma in infected people [1]. HBV is a small hepatotropic DNA virus belonging to the hepadnaviridae family, and preferentially infects hepatocytes via its receptor sodium taurocholate co-transporting polypeptide (NTCP) [2, 3]. After a mature HBV virus particle interacts with NTCP, the viral particle is internalized, and following the fusion of the viral and cellular membranes, the capsid is released into the cytoplasm and transported to the nucleus, where relaxed circular DNA (rcDNA) is repaired to form covalently closed circular DNA (cccDNA) with the assistance of host cell repair related proteins, including PCNA, RFC, Polδ, Fen-1, and LIG1 [4, 5]. cccDNA in the nucleus serving as a template is transcribed into subgenomic RNA and pregenomic RNA (pgRNA), which are both responsible for HBV life cycle and permit the persistence of HBV infection [6, 7]. cccDNA activity is regulated by epigenetic factors, virological factors, and immune-mediated factors [8]. However, there is still lack of methods to identify cccDNA-associated proteins directly in living cells.

CRISPR–Cas9 system is a powerful tool for genome editing [9]. Using an engineered nuclease-deficient Cas9 (dCas9) enables the repurposing of this system for targeting genomic DNA without cleaving DNA [10]. Previous studies have demonstrated that dCas9-single guide RNA (sgRNA) directly block the transcription of target genes by sterically hindering the RNA polymerase activity on the promoter or RNA polymerase processivity along a coding sequence [11]. Importantly, dCas9 can be fused with effectors to further enlarge its application potentials, such as transcriptional regulation, epigenetic modification, or genome repositioning [12–14]. Recently, CRISPR-dCas9 combined with ascorbate peroxidase 2 (APEX2) system offers a new approach for proximity-based labeling [15–17]. A comprehensive profile of DNA interaction with proteins at defined genomic loci has been identified by biotin labeling and liquid chromatography–mass spectrometry (LC–MS/MS), especially in living cells [16].

In this study, we describe a novel method to identify proteins associated with HBV cccDNA in host cells via dCas9-APEX2-mediated proximity labeling combined with LC–MS/MS. More importantly, we identified PRKDC as an effective activator of HBV cccDNA transcription through functional screening. Gene silencing by shRNA knockdown or inhibitor treatment significantly inhibited HBV cccDNA transcription in HBV-infected HepG2-NTCP cells and primary human hepatocytes (PHHs). In the nucleus, PRKDC could interact with POLR2A and POLR2B, the two largest subunits of RNA Pol II, and recruit Pol II to HBV cccDNA minichromosome dependent on its kinase function, as well as assist in phosphorylation at Ser5 and Ser2 of Pol II, thus promoting HBV transcription. Our studies suggest a novel mechanism for regulating HBV cccDNA transcription by host factors.

## Materials and methods

### Cell culture

HEK293T and HepG2 cells were obtained from ATCC. HepAD38 and HepG2-NTCP cell line were kindly provided by prof. Juan Chen (Chongqing Medical University, Chongqing, China). HepG2.2.15 cell line was kindly provided by prof. Yong Lin (Chongqing Medical University, Chongqing, China). HEK293T and HepG2 cells were maintained in DMEM (2022-03, Gibco, China) with 10% fetal bovine serum (FBS) (S711-001S, Lonsera, UY) and 100 U/ml penicillin–streptomycin (15140122, ThermoFisher, USA). HepG2.2.15 was maintained in RPMI-1640 medium (2021-03, Gibco, China) with 10% FBS and 100 U/ml penicillin–streptomycin. HepAD38 was grown in DMEM supplemented with 10% FBS, 400 μg/ml G418 (345810, Merck Millipore, Germany), and 100 U/ml penicillin–streptomycin. PHHs (LV-BIOTECH, Shenzhen, China) were maintained in PHH maintenance medium (LV-WEM001). All cells were cultured in a humidified incubator at 37 °C with 5% CO_2_.

### Plasmids, antibodies, and drugs

The plasmids PLH-sgRNA-2xMS2_no ccdb, pHAGE-EFS-MCP-APEX2-NLS and pHAGE-TO-dCas9-3XGFP were kindly provided by prof. Yun Liu (Fu Dan university, Shanghai, China). For RNAi experiments, shRNA plasmids were constructed by cloning the target sequences to the pLKO.1-puro vector. The small-guided RNA (sgRNA) expression vectors were cloned by inserting the annealed oligos into pLH-sgRNA1-2XMS2 (Addgene plasmid: 75389) at the BbsI site. All shRNA target and sgRNA sequences are shown in Supplementary Table S2.

Mouse anti-Cas9 (61957), mouse anti-RNA pol II (39097), mouse-anti-RNA pol II CTD phospho Ser2 (61984), mouse-anti-RNA pol II CTD phospho Ser5 (61986) were purchased from Active Motif. Rabbit anti-H3 (ab1791), rabbit anti-POLR2A (ab76123), mouse anti-PRKDC (ab32566), goat anti-PRKDC (ab168854) were purchased from Abcam. Normal rabbit IgG was purchased from Cell Signaling Technology. Mouse anti-GAPDH (A01020), mouse anti-β-ACTB (A01010) were purchased from Abbkine. Rabbit anti-POLR2B (A5928) was purchased from ABclonal. Mouse IgG was purchased from Merck Millipore. Rabbit anti-HBsAg (NB100-62652-0.1 ml) was purchased from NOVUS. SA-HRP (B110053) was purchased from Sangon Biotech. Mouse anti-HBc was kindly provided by prof. Xuefei Cai (Chongqing Medical University, Chongqing, China).

PRKDC inhibitor NU7026 (HY-15719) was obtained from MCE. Protease inhibitors (B14002) were purchased from Bimake.

### Virus production and infection

HBV particles were collected from the supernatants of HepAD38 cells. Cell debris was removed by centrifugation at 4000 rpm for 30 min at 4 °C and subsequently mixed with polyethylene glycol (PEG)8000 at final concentration of 6%. HBV Virion particles were concentrated at 4000 rpm for 41 min at 4 °C after gently rotating overnight at 4 °C. Pellet was re-dissolved in Opti-MEM (Gibco) at 100-fold concentration, viral titer was quantified by measuring HBV DNA with a qPCR assay. For infection, HepG2-NTCP cells were seeded in collagen-coated wells. The next day, HepG2-NTCP cells were treated with PRKDC shRNAs virus or NU7026 for 24 h, and cells were infected with HBV virus at an multiplicity of infection (MOI) of 1000 genome equivalents (Geq) per cell and were cultured in the presence of 4% PEG8000 and 2% DMSO for 24 h. Cells were washed three times with PBS and were maintained in infection medium for another 4 days; the medium was changed every 2 days. The supernatants and cells were collected for detection of HBV cccDNA transcripts.

### cccDNA isolation and quantitative analysis

cccDNA in HBV-infected cells were isolated by Hirt method with minor modification. Briefly, cells from six-well plate were lysed in 500 μl SDS lysis buffer (1% SDS, 50 mM Tris–HCl, pH 8.0, 10 mM EDTA, 150 mM NaCl) for 35 min. Then, the cell lysate was mixed with 125 μl 2.5 M KCl and incubated at 4 °C overnight. Mixture was centrifugated at 12,000 rpm for 20 min, the supernatant was collected and extracted by using phenol–chloroform two times. The final pellet was dissolved in 10 μl TE (10 mM Tris–HCl, pH 8.0, 1 mM EDTA) buffer.

For Taq-man probe qRT-PCR, the samples were digested with T5 Exonuclease (#M0663, New England Biolabs) before HBV cccDNA detection. And then, the treated samples were subjected to Taq-man probe qRT-PCR for cccDNA quantification. The primers and probe are listed in Supplementary Table S2.

Droplet digital PCR (ddPCR) was performed with the QX200^TM^ Droplet Digital PCR system according to the manufacturer’s instructions. Briefly, the samples were digested with T5 Exonuclease (#M0663, New England Biolabs) for 60 min at 37 °C, and then inactivated at 70 °C for 30 min. After mixing with primers, the samples were treated with UNG enzyme. Next, the droplets were transferred to a PCR plate for amplification with a cycle starting at 95 °C for 10 min, followed by 40 cycles of 94 °C for 30 s and 60 °C for 60 s, and a final cycle of 98 °C for 10 min. The droplets were analyzed on a QX200^TM^ droplet reader. The primers and probe are listed in Supplementary Table S2.

### Northern blotting

Total RNAs were isolated using the TRIzol reagent (Invitrogen). The extracted RNA was electrophoresed on 1.5% formaldehyde-agarose gel, and then was transferred to nylon membrane (11417240001, Roche, Germany). The following experiments were carried out according to DIG Northern Starter Kit (12039672910, Roche, Germany) manufacturer’s protocol. Finally, the membrane was exposed by using X-ray film.

### cccDNA-associated proteins isolation

Stable cell line to express sgRNA-2xMS2, MCP-APEX2, and dCas9 was established firstly, and then infected with HBV particles for 5 days. Proximity labeling was performed as described by Hung et al. [18]. Briefly, cells were treated with 500 μM biotin–phenol (A8011, APExBIO, America) for 30 min at 37 °C, hydrogen peroxide was added to the medium at final concentration of 1 mM and the cells were incubated for 1 min. Then, the reaction was immediately quenched by addition of quench buffer (10 mM sodium ascorbate and 5 mM Trolox). The cell pellets were resuspended using nuclear lysis buffer (50 mM Tris–HCl, pH 7.5, 500 mM NaCl, 0.4% SDS, 5 mM EDTA, 1 mM DTT, and 1× Protease inhibitors), and sonicated to yield fragments with an average length of 500–1000 bp. Supernatants were incubated with 50 μl High-Capacity Streptavidin Agarose Resin (20357, Thermo) overnight at 4 °C with rotation. The agarose resin was washed sequentially with wash buffer 1 (2% SDS), wash buffer 2 (0.1% sodium deoxycholate, 1% Triton X-100, 500 mM NaCl, 1 mM EDTA, 50 mM HEPES, pH 7.5), wash buffer 3 (250 mM LiCl, 0.5% NP-40, 0.5% sodium deoxycholate, 1 mM EDTA, 10 mM Tris–HCl, pH 8.0), and wash buffer 4 (50 mM Tris–HCl, pH 7.5, 50 mM NaCl). Finally, the agarose was resuspended in 30 μl 3× protein loading buffer supplemented with 2 mM biotin and 20 mM DTT, boiled at 95 °C for 10 min, and the supernatants were collected for further MS analysis to identify enriched proteins in samples with HBV cccDNA-targeted sgRNAs versus the samples with the sgGal4.

### Quantitative reverse transcription PCR (qRT-PCR)

Total RNAs were isolated using the TRIzol reagent (Invitrogen), and the first cDNA strand was synthesized with FastKing RT Kit (With gDNase) (TIANGEN, KR116-02). Real-time qPCR was then carried out through FastStart Essential DNA Green Master mix (Roche). The selective primers are listed in Supplementary Table S2.

### Chromatin immunoprecipitation (ChIP)

For ChIP experiments, 5 million cells were fixed with 1% formaldehyde for 10 min at room temperature. Cells were lysed at 25 °C for 5 min in lysis buffer (1% SDS, 10 mM EDTA and 50 mM Tris–HCl, pH 8.0, 1× Protease inhibitors, 20 mM sodium butyrate). The chromatin was sonicated to yield fragments with an average length of 300–700 bp, and then placed sheared chromatin on Ultracel-50K (UFC805096, Millipore, Germany) columns, spin at 4000×*g* for 3 min at 25 °C. Discarding the pass-through, and adding 1 ml IP buffer (10 mM Tris–HCl, pH 8.0, 1 mM EDTA, 0.5 mM EGTA, 50 mM NaCl, 5 mM sodium butyrate and 1× Protease inhibitors), spin at 4000×*g* for 7 min at 25 °C, repeat this step once. Transferring chromatin to a fresh 1.5 ml tube carefully and adjusting the volume to 1.1 ml using IP buffer. And then 50 μl of sample was taken out as input, and the rest was then subjected to immunoprecipitation using the indicated antibodies. Finally, the products of ChIP assays were detected by PCR. The selective primers are listed in Supplementary Table S2.

### Co-immunoprecipitation (Co-IP)

For Co-IP experiments, 6 million cells were lysed by RIP buffer (150 mM KCl, 25 mM Tris-pH, 7.4, 5 mM EDTA, 0.5 mM DTT, 0.5% NP40, 1× Protease inhibitors) on ice for 5 min. The cell lysates were sonicated to yield fragments with an average length around 1000 bp. The protein samples were then immunoprecipitated with the indicated antibodies overnight at 4 °C. The next day, 15 μl Dynabeads^TM^ Protein G beads were added to the sample and incubated 2 h at 4 °C. The beads were washed with RIP buffer for five times. Finally, the products were eluted by protein loading buffer.

### Western blotting

Protein samples were separated by SDS–PAGE, and then transferred to PVDF membrane, the membrane was blocked with 5% milk. Primary antibodies were diluted with QuickBlock^TM^ Western Primary Antibody Dilution Buffer (P0256, Beyotime), and incubated overnight at 4 °C. Following the incubation with the appropriate secondary antibodies, chemiluminescence was visualized (Millipore, USA).

### Determination of HBsAg or HBeAg

The supernatants from cell culture were collected, HBsAg or HBeAg levels were quantified by using ELISA kit from KHB (China) according to the manufacturer’s instructions.

### Immunofluorescence Staining

HBV-infected HepG2-NTCP or HepG2.2.15 cells were grown on coverslip. The cells were fixed in 4% paraformaldehyde for 10 min at room temperature, and then permeabilized with 0.5% Triton X-100 for 10 min. Blocking in 4% BSA for 1 h, the coverslip was incubated with the indicated antibody at 4 °C overnight. Washing the coverslip three times using PBS, cells were incubated with secondary antibodies conjugated with Alexa Fluor 488 or Alexa Fluor 594 for 1 h at room temperature. The nuclear staining was carried out by incubating with DAPI for 5 min. Finally, images were captured by using a confocal laser scanning microscope (LEICA).

### Statistical analysis

The results are shown as means of replicates. Statistical analyses were performed using Student’s *t*-test. Differences were considered significant when *P* < 0.05. All statistical analysis was performed by using R software.

## Results

### Systematic identification of cccDNA associated proteins

**Fig. 1:**
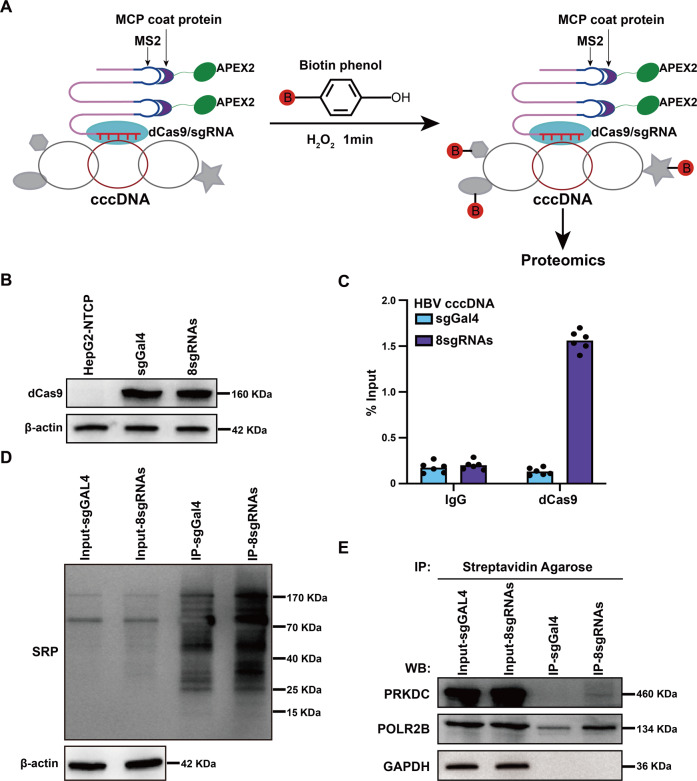
Systematic identification of cccDNA-associated proteins. **A** Schematic of the methodology to identify cccDNA-associated proteins. MCP MS2 coat protein. **B** Western blotting (WB) analysis with antibodies against dCas9. β-actin was used as a loading control. **C** The level of dCas9 associated with cccDNA was examined by ChIP-qPCR assay. Cross-linked chromatin was immunoprecipitated with specific anti-dCas9 antibody or corresponding IgG, followed by PCR quantification of HBV cccDNA using specific primers. **D** APEX2-mediated biotinylation of endogenous proteins associated with cccDNA, as detected by WB with streptavidin-HRP (SRP). **E** Mass spectrometry results were verified by immunoprecipitation and WB assays. HBV-infected cells were collected, lysates were immunoprecipitated with streptavidin agarose, and the products were separated by SDS–PAGE. GAPDH antibody was used as a negative control.

Exploring the factors responsible for the formation, maintenance, and transcriptional regulation of cccDNA could reveal potential targets for chronic infection treatments. Thus, to identify proteins associated with cccDNA, we combined a modified CRISPR system with peroxidase APEX2 [16] to target cccDNA in HBV-infected host cells (Fig. 1A). The MS2 RNA and MS2 coat protein (MCP) enabled the formation of a dCas9/sgRNA–MCP–APEX2 complex, which was capable of in-cell biotinylation of nearby proteins located within 20 nm in the presence of substrates, hydrogen peroxide, and biotin–phenol (Fig. 1A).

We designed eight sgRNAs to target HBV cccDNA, and sgRNA sgGal4 served as non-specific negative control. Stable cell lines expressing dCas9-sgGal4-APEX2 (labeled as ‘sgGal4’) and dCas9-8sgRNAs-APEX2 (labeled as ‘8sgRNAs’) were established first using HepG2-NTCP cells, and the expression of dCas9 was comparable between the sgGal4 and 8sgRNAs stable cell lines (Fig. 1B). When infected with HBV particles, we found that the secretion of HBeAg or HBsAg and the expression of HBV RNAs remained unchanged between these cell lines (Supplementary Fig. S1), which meant that these two stable cell lines did not change the accessibility to HBV particles or their expression of cccDNA. Next, we used chromatin immunoprecipitation (ChIP) assay to test whether the dCas9 could bind to cccDNA efficiently, and quantitative real-time PCR (qPCR) results using cccDNA-specific primers showed that dCas9 can be successfully recruited to cccDNA as compared with our sgGal4 control (Fig. 1C). HBV-infected cells were treated with hydrogen peroxide and biotin–phenol, and then lysed to enrich target proteins using streptavidin agarose resin. Streptavidin-HRP (SRP) blotting showed that the signals from 8sgRNAs immunoprecipitation (IP) products were stronger than those from sgGal4 IP products (Fig. 1D). Taken together, these results demonstrated that the method we established could successfully detect proteins associated with cccDNA.

Purified samples generated using the method above were then separated by SDS–PAGE, followed by mass spectrometry (MS) to identify specific enriched proteins in samples with cccDNA-targeted sgRNAs versus the samples with the sgGal4. We identified a total of 135 proteins enriched in 8sgRNAs IP products (Supplementary Table S1), including some previously reported cccDNA-associated proteins, such as PRMT1 and DDX5. We selected PRKDC, also known as DNA-PKcs, for further investigation because of its protein score, as it was ranked first among these 135 proteins. The mass spectrum of PRKDC is shown in Supplementary Fig. S2. Immunoblotting analysis demonstrated that PRKDC could be significantly enriched compared with negative control products, and POLR2B and glyceraldehyde-3-phosphate dehydrogenase (GAPDH) served as a positive control and negative control, respectively (Fig. 1E).

### Depletion of PRKDC inhibits HBV transcription

**Fig. 2:**
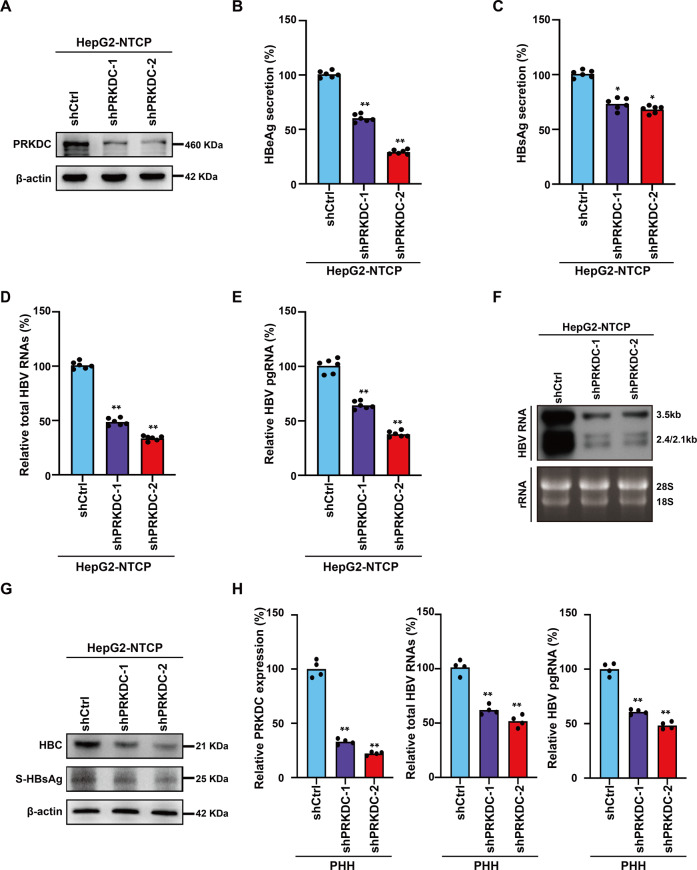
PRKDC silencing inhibits HBV transcription. **A** The knockdown efficiency was verified by WB analysis with antibodies against PRKDC. β-actin was used as a loading control. **B**, **C** PRKDC suppression decreased HBeAg (**B**) and HBsAg (**C**) secretion, as detected by ELISA kit. **D–F** The expressions of total HBV RNAs and 3.5-kb pgRNA were down regulated upon PRKDC disruption. HBV RNAs (**D**) and pgRNA (**E**) were analyzed by real-time PCR using specific primers and Northern blotting (**F**). Ribosomal RNAs (28S and 18S) served as loading controls. **G** HBsAg and HBc were analyzed by immunoblotting analysis. **H** PRKDC suppression in HBV-infected PHHs decreased total HBV RNAs and 3.5-kb RNA. *P < 0.05; **P < 0.01.

Previous studies have suggested that PRKDC participates in the regulation of transcription in various cancer cells [19, 20], and our MS results indicated that PRKDC was closely associated with cccDNA. Therefore, we hypothesized that PRKDC played a critical role in regulating HBV transcription. To test the roles of PRKDC in transcription regulation, we knocked down PRKDC gene expression by RNA interference (RNAi) with two independent short hairpin RNAs (shRNAs) in HepG2-NTCP cells (Fig. 2A), a widely used infection cell model to study HBV. We found no apparent changes in the hepatocyte status or cell cycle in PRKDC-depleted cells (Supplementary Fig. 3A–C), indicating that the alterations in transcription were not due to biased cell cycling or status. The amounts of HBeAg and HBsAg in cell culture supernatants were decreased significantly upon PRKDC knockdown (Fig. 2B, C). Moreover, HepG2-NTCP cells expressing shCtrl and shPRKDC showed a decrease in total HBV RNAs and pgRNA as detected by real-time PCR (Fig. 2D, E). Consistently, Northern blotting analysis confirmed that PRKDC deletion in HBV-infected HepG2-NTCP cells decreased the production of 3.5, 2.4, and 2.1-kb HBV RNAs (Fig. 2F). Western blot analysis using a specific antibody for HBc or HBsAg also confirmed that the depletion of PRKDC could reduce the viral RNA and protein accumulation (Fig. 2G and Supplementary Fig. S4). Based on immunofluorescence analysis, we observed strong signal reductions for HBc in PRKDC-depleted cells (Supplementary Fig. S5A). Similarly, PRKDC knockdown in HBV-infected PHHs decreased total HBV RNAs, 3.5-kb RNA (Fig. 2H). Moreover, the effect of PRKDC depletion on HBV transcription was further investigated in HepG2.2.15 cells, in which HBV RNA synthesis regulation depends on its own promoter. The data showed that PRKDC knockdown decreased HBeAg secretion, total HBV RNAs levels, and pgRNA levels, as well as HBc protein levels in HepG2.2.15 cells (Supplementary Fig. S6A–C). Taken together, these findings indicated that PRKDC plays potentially vital roles in regulating HBV transcription.

### NU7026, an inhibitor of PRKDC, reduced cccDNA transcription

**Fig. 3:**
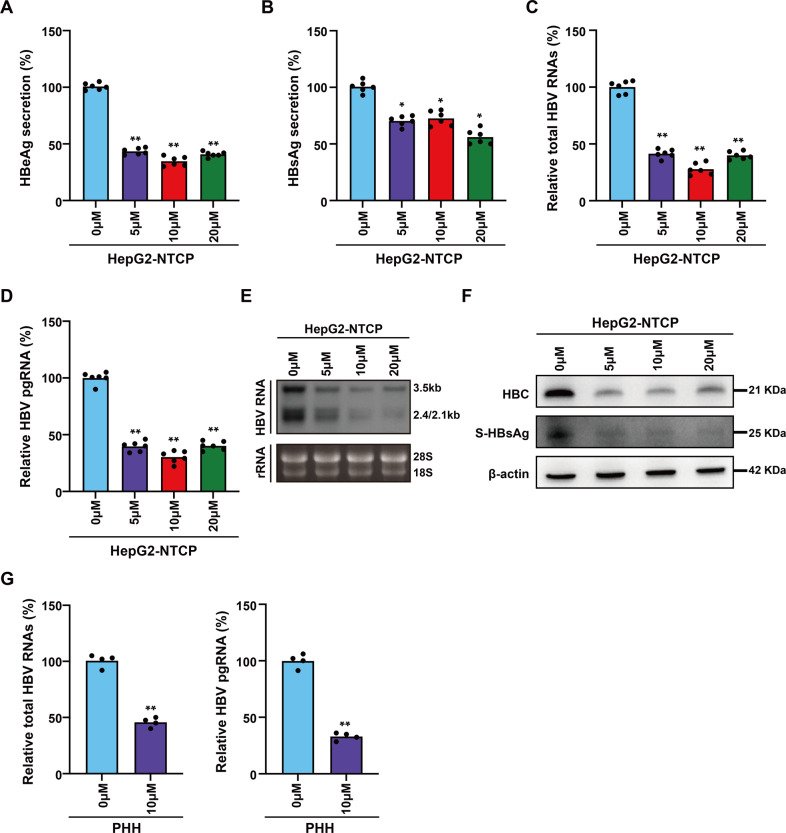
NU7026 reduced cccDNA transcription. HBV-infected HepG2-NTCP cells or PHHs were treated with a concentration gradient of NU7026, and the concentrations of HBeAg (**A**) and HBsAg (**B**) in cell culture supernatants were detected by ELISA kit. Total HBV RNA (**C**) and pgRNA (**D**) transcriptions were analyzed by real-time PCR using specific primers and Northern blotting (**E**). Ribosomal RNAs (28 S and 18 S) served as loading controls. HBsAg and HBc were detected by immunoblotting analysis (**F**). **G** NU7026 treated in HBV-infected PHHs decreased total HBV RNAs and 3.5-kb RNA. *P < 0.05; **P < 0.01.

To further investigate the functional roles of PRKDC on cccDNA transcription, we used 2-(morpholin-4-yl)-benzo[h]chomen-4-one (NU7026), a competitive and highly selective inhibitor of DNA-PKcs [21], to treat HBV-infected HepG2-NTCP cells. There were no apparent changes in the cell cycle of HepG2-NTCP cells in a concentration gradient treatment (Supplementary Fig. S3D). However, the secretion of HBeAg and HBsAg were decreased significantly upon NU7026 treatment (Fig. 3A, B). Moreover, the accumulation of HBV RNAs and pgRNA was inhibited significantly (Fig. 3C, D), and Northern blotting analysis showed that the production of 3.5, 2.4, and 2.1-kb HBV RNAs was decreased in HBV-infected HepG2-NTCP cells by inhibitor treatment (Fig. 3E). Based on Western blot and immunofluorescence analysis, we observed strong reductions of HBc and HBsAg in NU7026 treated cells (Fig. 3F and Supplementary Fig. S5B). Moreover, PRKDC knockdown in HBV-infected PHHs decreased total HBV RNAs, 3.5-kb RNA (Fig. 3G). Consistently, the decrease of HBeAg secretion, total HBV RNAs, pgRNA, and HBc proteins was also observed in HepG2.2.15 cells (Supplementary Fig. S7A–C), further verifying the regulatory functions of PRKDC in HBV cccDNA transcription.

### PRKDC is dispensable for the formation of cccDNA

**Fig. 4:**
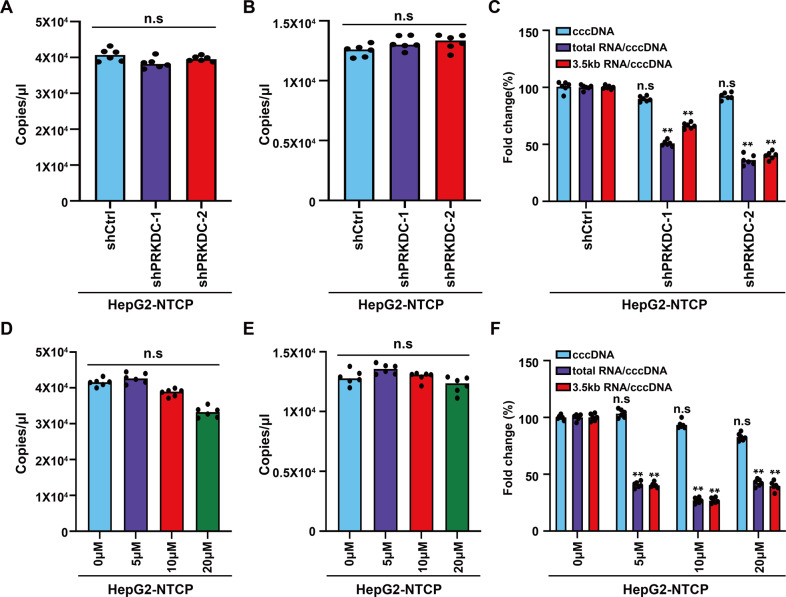
PRKDC is dispensable for the formation of cccDNA. HBV cccDNA was extracted at day 5 post HBV infection using the Hirt method. Real-time PCR analysis was performed using cccDNA-specific primers upon shRNA knockdown (**A**) or NU7026 treatment (**D**). Droplet digital PCR (ddPCR) was also carried out to detect the numbers of cccDNA after PRKDC knockdown (**B**) or inhibitor treatment (**E**). The decrease in total HBV RNAs or pgRNA expression was calculated as the ratio of normalized HBV RNAs or pgRNA to cccDNA after PRKDC knockdown (**C**) or inhibitor treatment (**F**). **P < 0.01; n.s. not significant.

HBV DNA is converted from its relaxed circular form to closed covalent circular DNA (cccDNA) after transport to the nucleus, which served as a template for all viral RNA transcription. It has been reported that the non-homologous end joining (NHEJ) DNA repair pathway participates in the formation of cccDNA [5, 22, 23]. As one of the core components of DNA–PK complex, the activation of PRKDC is required for the completion of repair. To rule out that the decrease in HBV transcription was not caused by the inhibition of cccDNA formation, we extracted cccDNA from HBV-infected HepG2-NTCP cells using Hirt method. qPCR analysis results showed that the number of cccDNA remained unchanged upon shRNA or NU7026 treatment (Fig. 4A, D). Consistently, Droplet digital PCR (ddPCR), which can quantify cccDNA content more accurately, detected no changes in the cccDNA number after treatment with shRNA (Fig. 4B) or inhibitor (Fig. 4E). More importantly, PRKDC knockdown or NU7026 treatment decreased the ratios of total RNA/cccDNA and 3.5-kb RNA/cccDNA (Fig. 4C, F). Thus, the decrease in HBV RNAs may have been due to transcriptional regulation by PRKDC.

### PRKDC interacts with POLR2A and POLR2B, and associates with cccDNA

The above findings encouraged us to investigate how PRKDC regulates HBV cccDNA transcription. Our MS results demonstrated that POLR2B, the second largest subunit of RNA Pol II, could be enriched significantly compared with our negative control (Fig. 1F), and cccDNA was transcribed by the host RNA Pol II [24]. Therefore, we hypothesized that PRKDC could facilitate cccDNA transcription through interacting with Pol II.

**Fig. 5:**
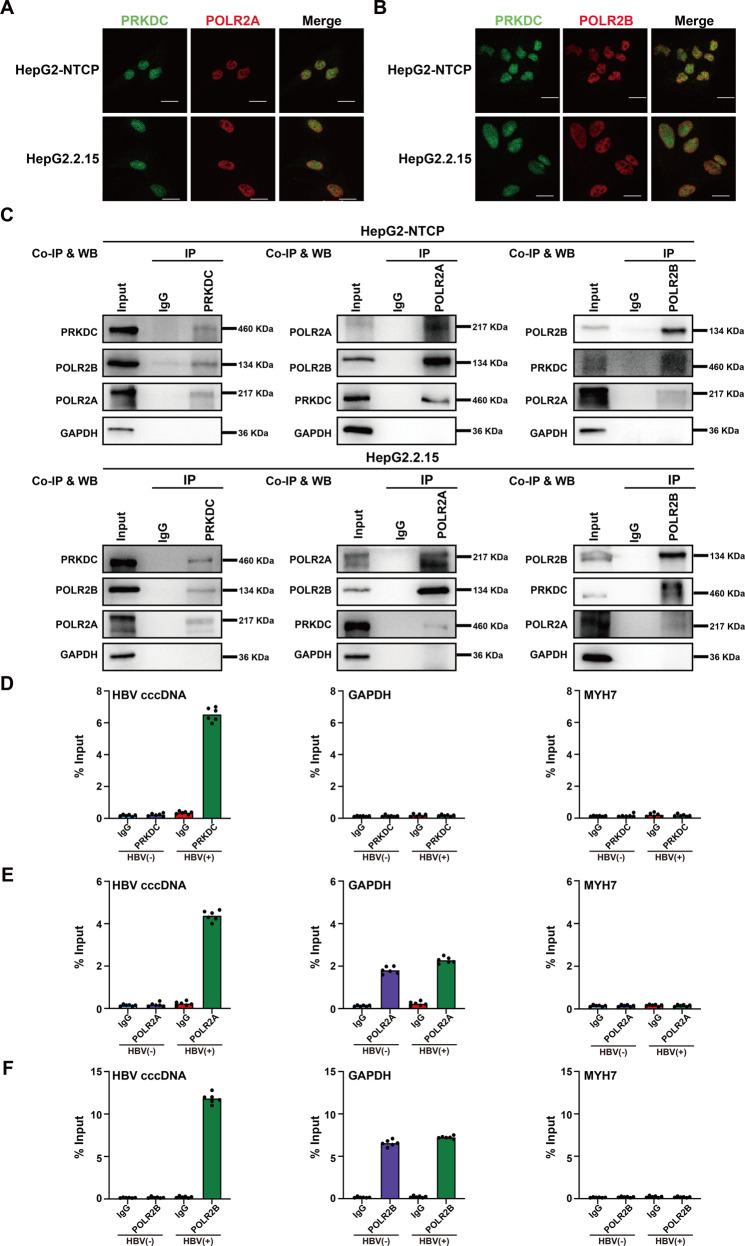
PRKDC interacts with POLR2A and POLR2B, and associates with cccDNA. **A, B** Immunofluorescent staining demonstrates partial colocalization of PRKDC (green) and POLR2A (**A**) or POLR2B (**B**) in HBV-infected HepG2-NTCP and HepG2.2.15 cells. Scale bar, 10 μm. **C** Endogenous co-immunoprecipitation with PRKDC (left), POLR2A (middle), and POLR2B (right) was carried out in HBV-infected HepG2-NTCP and HepG2.2.15 cells using indicated antibodies and blotted with specific antibodies. **D–F** Cross-linked chromatin from HBV-infected or non-HBV-infected HepG2-NTCP cells was immunoprecipitated with PRKDC (**D**), POLR2A (**E**), and POLR2B (**F**), and the corresponding IgG was used as a control, followed by PCR quantification of HBV cccDNA and the promoter of *GAPDH* and *MYH7* using specific primers. The results are displayed as the percentage of input.

To investigate the interactions between PRKDC and Pol II, we chose POLR2A and PLOR2B, the largest and second largest subunits of Pol II, respectively, for further studies. Our immunofluorescence data indicated that both POLR2A and POLR2B were partially colocalized with PRKDC in the nucleus of HBV-infected HepG2-NTCP and HepG2.2.15 cells (Fig. 5A, B). We then examined whether PRKDC physically associated with POLR2A or POLR2B in vivo using a reciprocal Co-immunoprecipitation (Co-IP) assay. Co-IP assays were carried out using HBV-infected HepG2-NTCP and HepG2.2.15 cells. The data revealed that POLR2A and POLR2B physically associated with PRKDC in both cell models (Fig. 5C and Supplementary Fig. S8).

HBV cccDNA serves as a template for the transcription of all viral RNAs. Next, we used chromatin immunoprecipitation (ChIP) assay to test whether PRKDC, POLR2A, and POLR2B could associate with cccDNA. The housekeeping gene *GAPDH* is highly expressed in HepG2-NTCP cells, which was used as a control for activated transcription. Myosin Heavy Chain 7 (MYH7), however, is expressed predominantly in normal human ventricles, with almost no expression in HepG2-NTCP cells, the promoter of *MYH7* was used as a control for repressed transcription. Our ChIP results demonstrated that PRKDC, POL2A, and POLR2B were all recruited onto cccDNA upon HBV infection (Fig. 5D–F). Further analysis revealed that PRKDC could not bind the promoter of *GAPDH* or *MYH7*, regardless of whether the cells were infected with HBV. However, the status of POLR2A and POLR2B at the promoter of *GAPDH* was relatively stable (Fig. 5D–F), implying that PRKDC could, to some extent, preferentially bind to cccDNA.

### PRKDC knockdown and NU7026 treatment inhibit the binding of POLR2A and POLR2B to HBV cccDNA

**Fig. 6:**
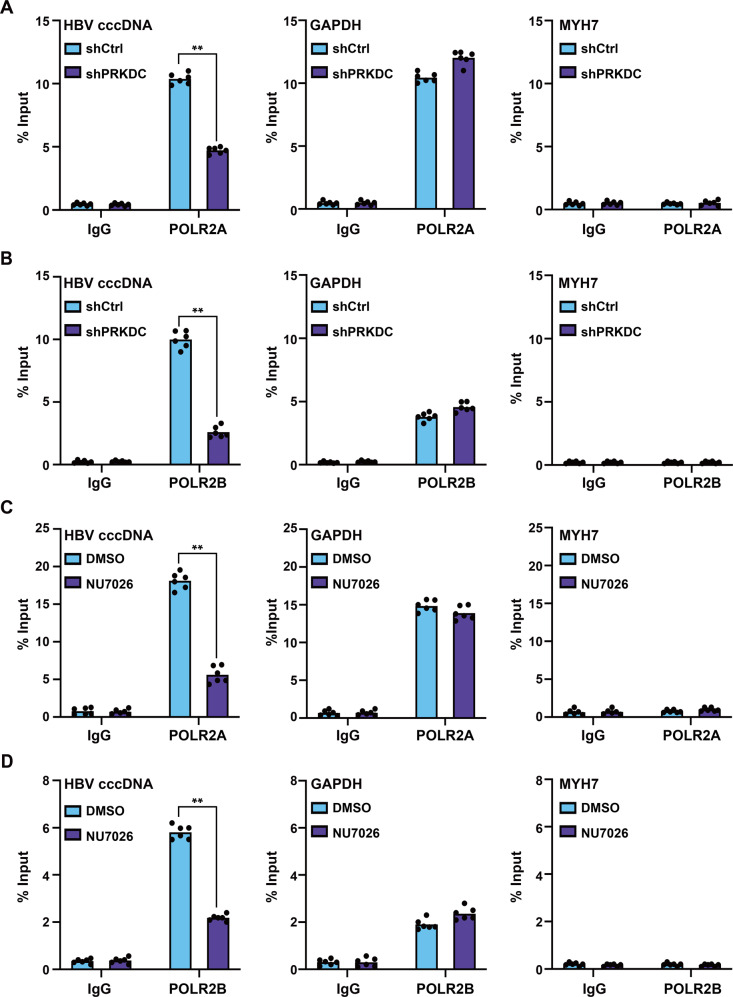
PRKDC knockdown and NU7026 treatment inhibits the binding of POLR2A and POLR2B to HBV cccDNA. HBV-infected HepG2-NTCP cells were treated with PRKDC shRNAs or NU7026, and then cross-linked with 4% formaldehyde solution, sonicated, and immunoprecipitated with indicated antibodies. Levels of POLR2A (**A**) or POLR2B (**B**) associated with HBV cccDNA or the *GAPDH* or *MYH7* promoter upon PRKDC knockdown were determined by qPCR. The effect of NU7026 treatment on the recruitment of POLR2A (**C**) or POLR2B (**D**) to cccDNA or the *GAPDH* or *MYH7* promoter was examined by ChIP-qPCR. The results are displayed as the percentage of input. **P < 0.01.

To further investigate the molecular mechanism of PRKDC in cccDNA transcription regulation, we used cccDNA ChIP-qPCR to test whether PRKDC depletion could affect the binding of host RNA Pol II to the cccDNA minichromosome. HBV-infected HepG2-NTCP cells were treated with shRNA virus and NU7026, respectively, the chromatin was sonicated, and then immunoprecipitated with specific antibodies. As expected, the enrichment of POLR2A and POLR2B on cccDNA was markedly decreased upon PRKDC knockdown (Fig. 6A, B). However, as a control, the binding levels of POLR2A and POLR2B at the promoter of *GAPDH* were relatively stable, and without any binding to the promoter of *MYH7* (Fig. 6A, B), consistent with the binding preference for cccDNA. More importantly, NU7026 treatment also decreased the enrichment of POLR2A and POLR2B on cccDNA (Fig. 6C, D). Together, these results indicated that cccDNA-bound Pol II was an active marker of cccDNA transcription and its association with cccDNA needed the assistance of PRKDC, in a protein kinase-dependent manner.

### cccDNA-associated Pol II CTD Ser2 and Ser5 phosphorylation modifications decreased upon PRKDC deletion or NU7026 treatment

**Fig. 7:**
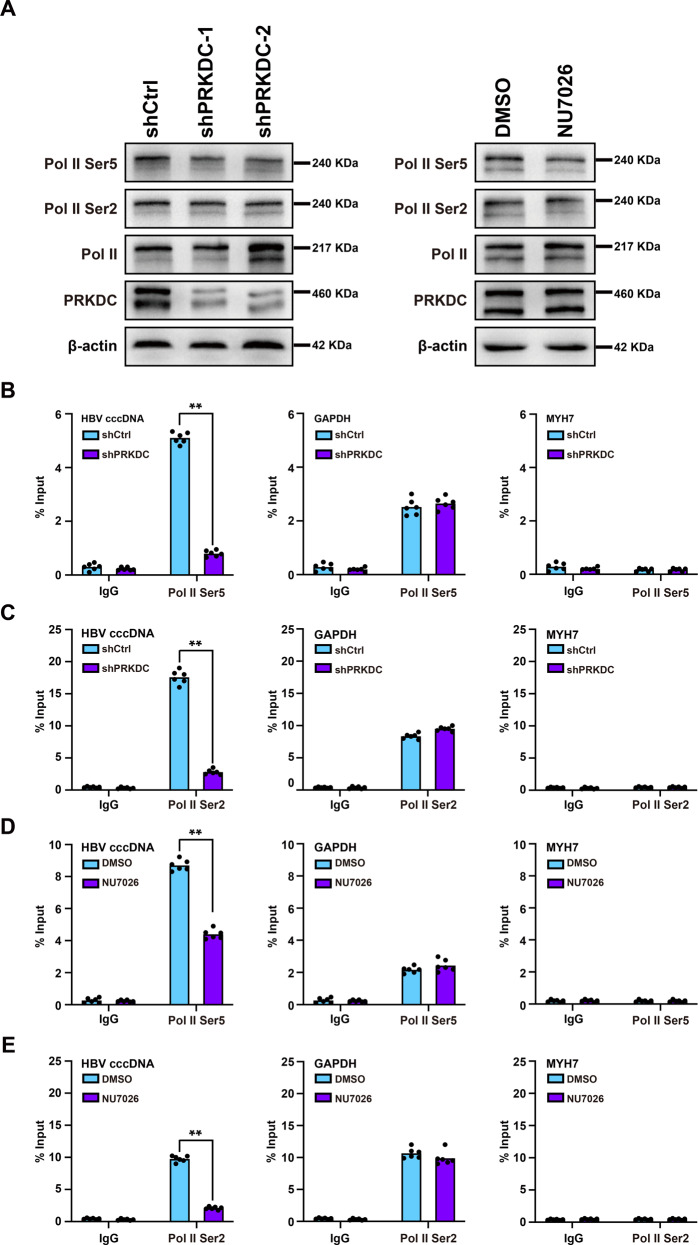
cccDNA-associated Pol II CTD Ser2 and Ser5 phosphorylation modifications decrease upon PRKDC depletion or NU7026 treatment. **A** Western blot analysis with antibodies against specified proteins. β-actin was used as a loading control. **B**, **C** Effects of PRKDC knockdown on the recruitment of Pol II Ser5 or Pol II Ser2 to cccDNA. The level of Pol II Ser5 (**B**) or Pol II Ser2 (**C**) associated with HBV cccDNA or the *GAPDH* or *MYH7* promoter was analyzed by ChIP assay with the indicated antibodies. **D**, **E** NU7026 treatment in HBV-infected HepG2-NTCP cells correlated with decreased Pol II Ser5 and Pol II Ser2 binding. The level of Pol II Ser5 (**D**) or Pol II Ser2 (**E**) associated with HBV cccDNA or the *GAPDH* or *MYH7* promoter was analyzed by ChIP assay. The results are displayed as the percentage of input. **P < 0.01.

The C-terminal repeat domain (CTD) of the largest subunit of RNA Pol II, POLR2A, is composed of tandem heptad repeats with the consensus sequence Tyr1-Ser2-Pro3-Thr4-Ser5-Pro6-Ser7, and RNA Pol II phosphorylated at Ser2 is enriched over the gene body and is associated with transcriptional elongation. RNA Pol II Ser5 phosphorylation, however, is often confined to promoter regions and is necessary for the initiation of transcription. Moreover, it has been reported that DNA-dependent protein kinase (DNA-PK) phosphorylates RNA Pol II [25, 26]. Thus, PRKDC may regulate the levels of phosphorylation modifications at Ser5 and Ser2 associated with HBV cccDNA. Antibodies specific for Pol II Ser2 and Pol II Ser5 were detected by ChIP-qPCR (Supplementary Fig. S9), and immunoblotting analysis indicated that the global levels of RNA Pol II Ser5 or Ser2 phosphorylation remained roughly unchanged (Fig. 7A).

Next, we used ChIP assay to further analyze whether the enrichment of RNA Pol II Ser5 or Ser2 phosphorylation had changed among cccDNA. The data indicated that PRKDC depletion by shRNA knockdown or NU7026 treatment significantly decreased the enrichment of RNA Pol II Ser5 on cccDNA in HBV-infected HepG2-NTCP cells, without any effect on the recruitment of RNA Pol II Ser5 to the promoter of *GAPDH* or *MYH7* (Fig. 7B, D).

Phosphorylation at Ser5 of the Pol II CTD through the cyclin-dependent kinase 7 (CDK7) subunit helps to establish transcription pausing. Transcription elongation, however, requires phosphorylation at Ser2 of Pol II CTD and some other related proteins [27]. Therefore, we hypothesized that the decreased enrichment of RNA Pol II Ser5 to cccDNA may lead to lower levels of RNA Pol II Ser2 to cccDNA. To test this hypothesis, cccDNA ChIP assay was carried out to detect the enrichment of RNA Pol II Ser2 associated with cccDNA, and qPCR analysis found that PRKDC knockdown or NU7026 treatment significantly decreased the recruitment of RNA Pol II Ser2 to cccDNA (Fig. 7C, E). Taken together, these data suggested that PRKDC plays critical roles in the regulation of the phosphorylation of RNA Pol II associated with cccDNA.

## Discussion

In this study, we developed a novel method based on CRISPR-dCas9 and APEX2-mediated proximity labeling system to isolate the proteins interacting with HBV cccDNA in host cells. We showed that this CRISPR-dCas9-APEX2 system could specifically enrich the proteins associated with cccDNA in their native states and be used to successfully identify cccDNA-binding proteins via LC–MS/MS and immunoblot analysis. Subsequently, we confirmed that PRKDC was a key transcriptional regulator that promoted the transcription of HBV cccDNA. Mechanismly, PRKDC was coupled directly to RNA Pol II to facilitate phosphorylation at the CTD residue Serine-5 (Ser5) and Serine-2 (Ser2).

Histone modifications of the HBV cccDNA minichromosome play critical roles in transcription regulation. Hypoacetylation of the cccDNA-associated H3 and H4 histones, which is mediated by HDAC1, is associated with low HBV replication both in vitro and in vivo [28]. Consistently, HDAC11-mediated deacetylation among H3K9ac and H3K27ac also inhibits HBV transcription and replication in HBV-transfected and HBV in vitro infection systems [29]. Similarly, SIRT3 can increase the recruitment of the histone methyltransferase suppressor of variegation 3–9 homolog 1 to cccDNA and decrease the recruitment of SET domain containing 1A, leading to a marked increase in trimethyl-histone H3 (Lys9) and a decrease in trimethyl-histoneH3 (Lys4) on cccDNA, thus suppressing the transcription of cccDNA [30]. Besides histone deacetylation, the protein arginine methyltransferase PRMT5 was identified to be an effective restrictor of HBV transcription and replication through triggering symmetric dimethylation of arginine 3 on H4 on the cccDNA minichromosome. This involved an interaction with the HBV core protein and the Brg1-based human SWI/SNF chromatin remodeler [31]. Protein arginine methyltransferase 1 (PRMT1), SET domain bifurcated histone lysine methyltransferase 1 (SETDB1), and structural maintenance of chromosomes 5/6 (SMS5/6) also interact with cccDNA to silence HBV cccDNA transcription [32–34].

However, recent chromosome conformation capture (3C) studies have shown that cccDNA preferentially establishes contacts with the host DNA at active chromatin regions. Hi-C, viral DNA capture (CHi-C), or 3C-high-throughput genome-wide translocation sequencing (3C-HTGTS) in primary human hepatocytes or cellular models of HBV infection have shown that HBV contacts preferentially with CpG islands (CGIs) enriched in Cfp1, a factor that is often associated with highly expressed genes [35, 36]. Moreover, 3C has been used to identify potential interactions of cccDNA and cellular chromatin through rcccDNA transfection in hepatoma cells and has been used to demonstrate that cccDNA is specifically associated with human chromosome 19p13.11 region. This region contains a highly active enhancer element that is mediated by cellular transcription factor Yin-Yang 1 (YY1) and viral protein HBx [37]. More importantly, circularized chromosome conformation capture (4C) sequencing analysis have revealed that transcriptionally inactive cccDNA preferentially accumulates at specialized areas, including regions close to chromosome 19 (chr.19). Activation of the cccDNA is apparently associated with its re-localization from a pre-established heterochromatin hub to transcriptionally active regions and is mediated by HBx and the SMC5/6 complex [38]. Therefore, it is still necessary to identify proteins associated with cccDNA in vivo, which are responsible for the active regulation of HBV cccDNA transcription.

Targeting HBV cccDNA using the CRISPR-Cas9 gene editing system is a potentially curative strategy for chronic hepatitis B; yet this system inevitably targets integrated HBV DNA and induces double-strand breaks (DSBs) in the host genome, which increase the risk of genomic rearrangement and damage [39]. dCas9 is an engineered nuclease-deficient Cas9 that can allow the repurposing of this system for targeting genomic DNA without cleaving it [10]. APEX2 is an engineered ascorbate peroxidase and catalyzes a reaction using biotin–phenol to generate radicals and tag endogenous proteins proximal to APEX2, allowing their subsequent enrichment using streptavidin beads and identification by mass spectrometry [40, 41]. The APEX2 protein coupled to the CRISPR system via the MS2 coat protein is capable of proximity labeling at a predefined genomic locus [16, 42]. Thus, combining CRISPR-dCas9 and APEX2 could help us identify cccDNA-associated proteins in living cells. Based on functional screening, we found that PRKDC knockdown or NU7026 treatment significantly reduced total HBV RNAs and 3.5-kb pgRNA, as well as the secretion of HBsAg or HBeAg in HBV-infected HepG2-NTCP cells.

PRKDC, as a member of the phosphatidylinositol 3-kinase-related kinase (PIKK) family, is best known as a central component of NHEJ that serves to repair DNA DSBs [43]. DSBs are initially recognized by the Ku70/80 heterodimer, which then acts as a recruitment platform for the rest of the NHEJ proteins [44]. In particular, PRKDC binding to the Ku–DNA complex acquires kinase activity at several sites by autophosphorylation or allosteric regulation, allowing further recruitment and activation of various repair proteins, including Artemis, X-ray cross complementing protein4 (XRCC4), XRCC4-like factor (XLF), and other factors [45]. Biochemical and structural analyses have indicated that a DNA-dependent protein kinase (DNA–PK) complex is central to the DSB repair system. Previous studies have found that the NHEJ pathway participates in the formation of cccDNA; thus, we first wanted to test whether PRKDC knockdown or NU7026 treatment could affect the formation of cccDNA, which may lead to a decrease of HBV cccDNA transcripts. cccDNA was extracted from HBV-infected HepG2-NTCP cells using Hirt method. qPCR analysis and ddPCR results showed that the number of cccDNA remained unchanged upon shRNA or NU7026 treatment. The cccDNA in host cell nucleus is converted from rcDNA and double-stranded linear DNA (dslDNA) [46]. However, rcDNA is the main source of cccDNA, and the conversion from rcDNA involves multiple steps: firstly, tyrosyl-DNA-phosphodiesterase 2 (TDP2)-mediated release of virial polymerase [47] and removal of RNA primer from the positive strand by some yet unknown enzymes, which might lead to the formation of protein-free (PF)-rcDNA; and the next, redundant sequences from the negative strand were cleavaged by endonuclease 1 (FEN1) [48]; and then, the positive strand was repaired by a series of polymerases [49, 50]; lastly, the strands were ligated by DNA ligase 1 and 3 [5]. The dslDNA occupies small fractions of HBV DNA, which is the dominant substrate for integration into the host genome [51, 52], can also be circularized by the NHEJ DNA repair pathway into cccDNA-like molecules [52]. Therefore, PRKDC knock down or inhibitor NU7026 treatment has little effect on the formation of cccDNA. This data indicated that PRKDC may participate in the regulation of HBV cccDNA transcription.

It is known that the formation of a transcription complex requires the synergistic action and functional activity of auxiliary factors, which are phosphorylated by PRKDC at definite sites, including TRIM28, Sp1, and USF-1 [53–55]. PRKDC can drive cancer progression by interacting with androgen receptor or estrogen receptor to mediate transcriptional regulation [19, 56]. Additionally, since PRKDC phosphorylates γH2AX during the DNA damage response (DDR), it is also required for Pol II pause, release, and elongation [53]. Our MS results showed that PRKDC and POLR2B could be enriched significantly compared to negative control products, which was further validated by Western blotting. We then verified that PRKDC could physically associate with Pol II by using immunofluorescence and reciprocal Co-IP. Furthermore, cccDNA ChIP-qPCR showed that both PRKDC and Pol II could associate with HBV cccDNA. More importantly, PRKDC knockdown or NU7026 treatment significantly decreased the enrichment of Pol II Ser5 phosphorylation on cccDNA, which may have caused the transcription initiation of HBV cccDNA transcripts to be affected (Supplementary Fig. S10).

In conclusion, this study provided a novel approach for investigating HBV cccDNA interaction with host cell proteins and revealed a key mechanism for regulating the transcriptional activity of HBV cccDNA. This study suggested that PRKDC might be a potential target for HBV treatment.

## Supplementary information

checklist-CDDIS-21-5071-T

Supplyinformation

western blot raw data

## Data Availability

The data that support the findings of this study are contained within the article and the supporting information. Additional supporting data are available from the corresponding authors on request. We have submitted the raw MS data to ProteomeXchange via the PRIDE Archive.
