# Supplementary material for: PRKDC promotes hepatitis B virus transcription through enhancing the binding of RNA Pol II to cccDNA
Source: Cell Death Dis. 2022 Apr 25;13(4):404. doi: 10.1038/s41419-022-04852-3 (PMC9038722; doi:10.1038/s41419-022-04852-3)
Supplement: Supplementary file 2 — Supplyinformation [file 41419_2022_4852_MOESM2_ESM.pdf]

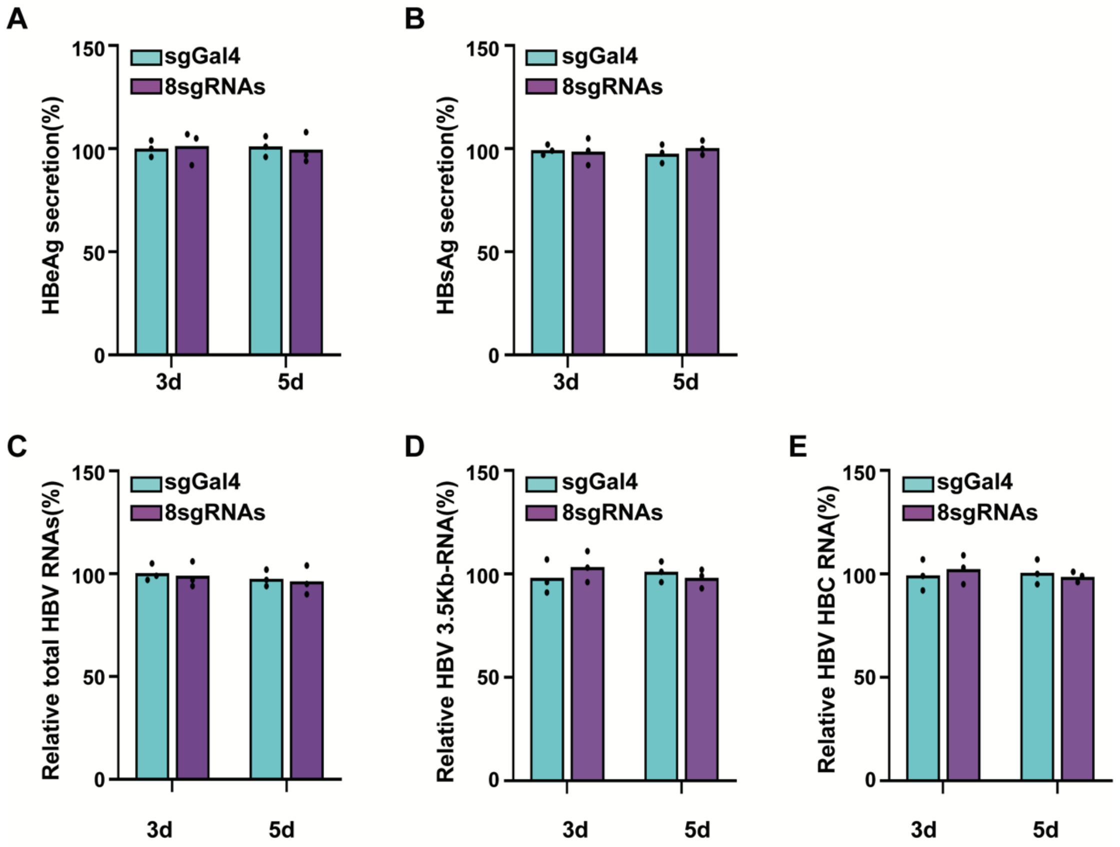

Figure S1. Stable cell lines denoted as sgGal4 and 8sgRNAs occupied same infection ability. Stable cell lines were infected with 1×10^3 genome equivalents/cell of HBV particles for 24 hours. Cells were harvested for evaluating transcription at indicated time points. The secretion of HBeAg (**A**) and HBsAg (**B**) in supernatant was detected using ELISA assay in HBV infected cells. HBV RNAs was analyzed by real-time PCR using specific primers (**C-E**).

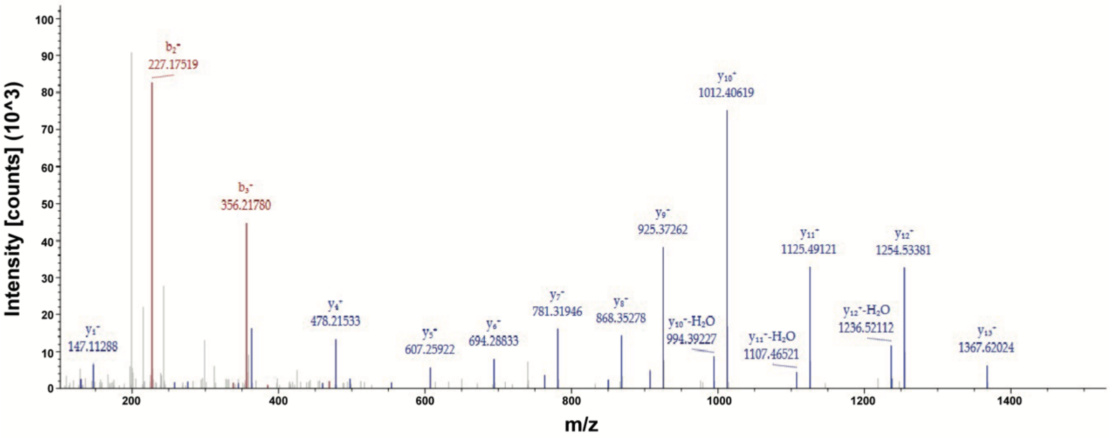

Figure S2. MS spectral identification of cccDNA associated proteins. The products were separated by SDS-PAGE followed by Mass Spectrometry (MS) analysis, PRKDC was selected as example.

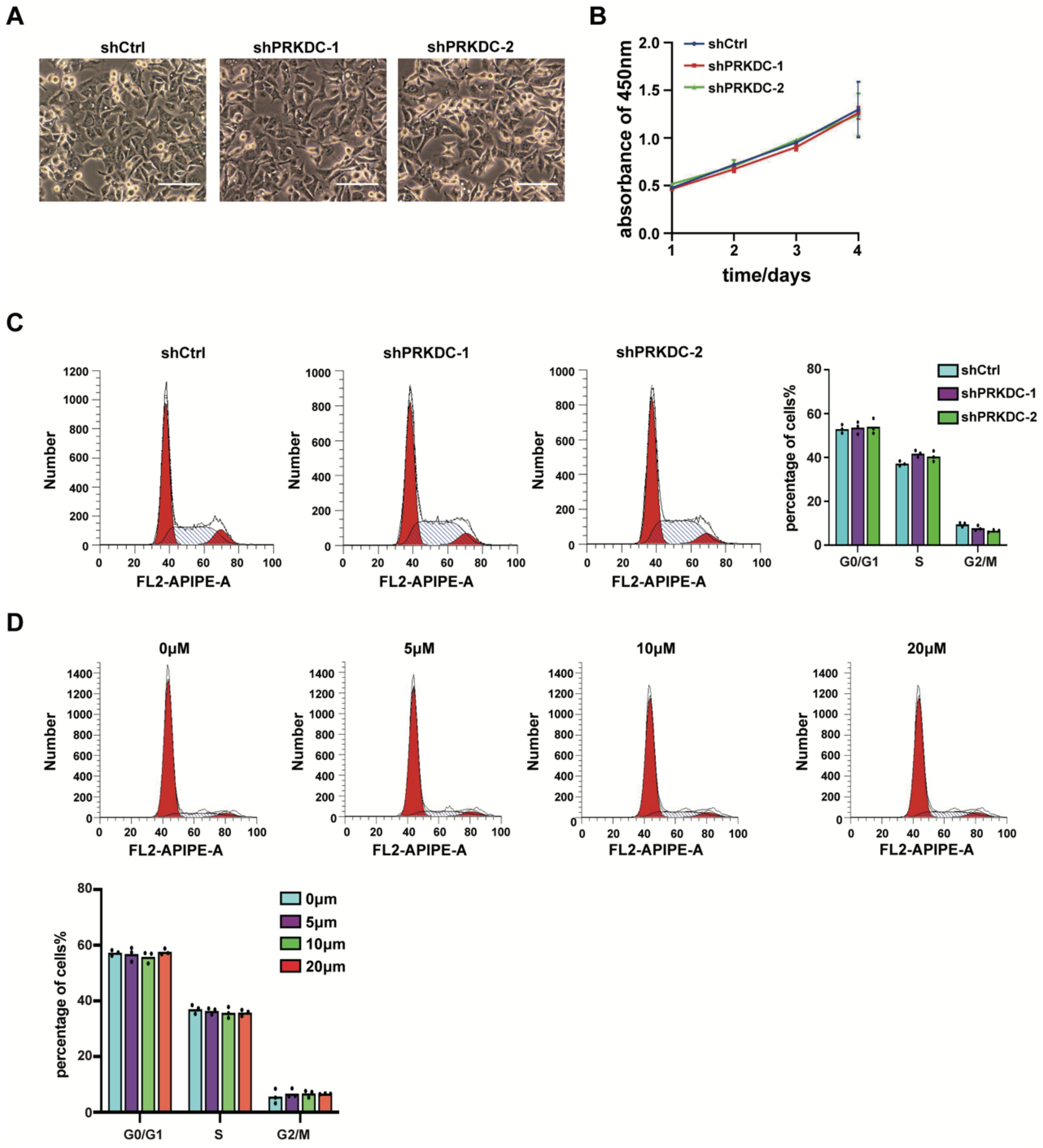

Figure S3. PRKDC knockdown or NU7026 treatment did not affect cell cycle and proliferation of HepG2-NTCP. (A) Bright field observation of cell status. The scale bar represents 50 μm. (B) Effects of PRKDC depletion on the cell proliferation were determined by the MTT assay. (C) Cell cycle analysis of shRNAs treated HepG2-NTCP cells by flow cytometry. (D) Cell cycle analysis of NU7026 treated HepG2-NTCP cells by flow cytometry.

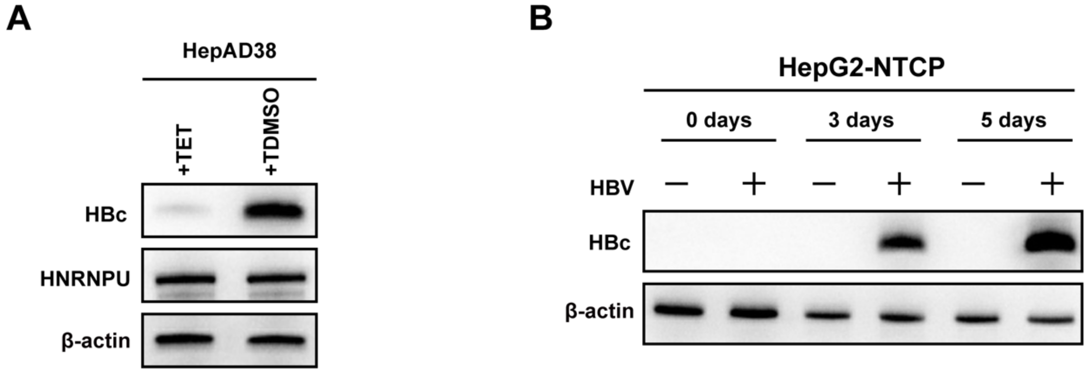

Figure S4. The specificity of HBc antibody was verified by western blot analysis. (A) The HepAD38 cell line was treated with Tetracycline (TET) and DMSO respectively for two weeks, the expression of HBc were analyzed by western blotting. HNRNPU and β-actin as loading controls. (B) HepG2-NTCP cells were infected with or without 1×10^3 genome equivalents/cell of HBV particles in the presence of 4% PEG8000 for 24h, respectively. the expression of HBc were analyzed by western blotting. β-actin as loading control.

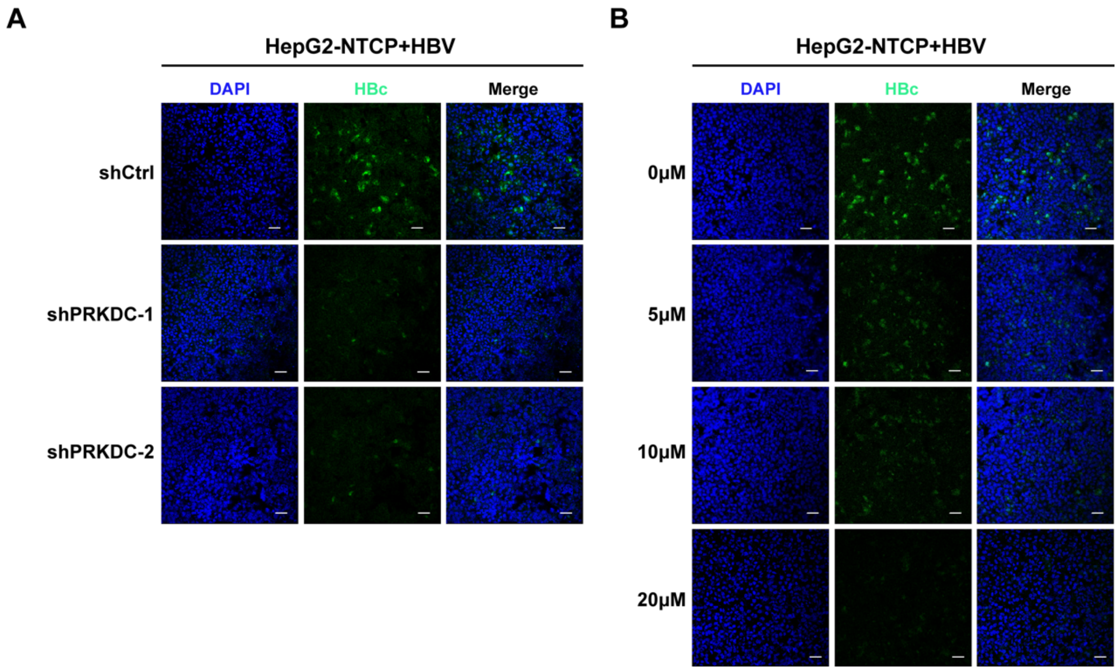

Figure S5. PRKDC knockdown or NU7026 treatment in HBV-infected HepG2-NTCP inhibited HBc expression. HepG2-NTCP cells were seeded in collagen-coated cover glass, and then transduced with lentivirus expressing the PRKDC knockdown shRNAs (A) or treated with NU7026 (B) for indicated days, the expressions of HBc were analyzed by immunofluorescence.

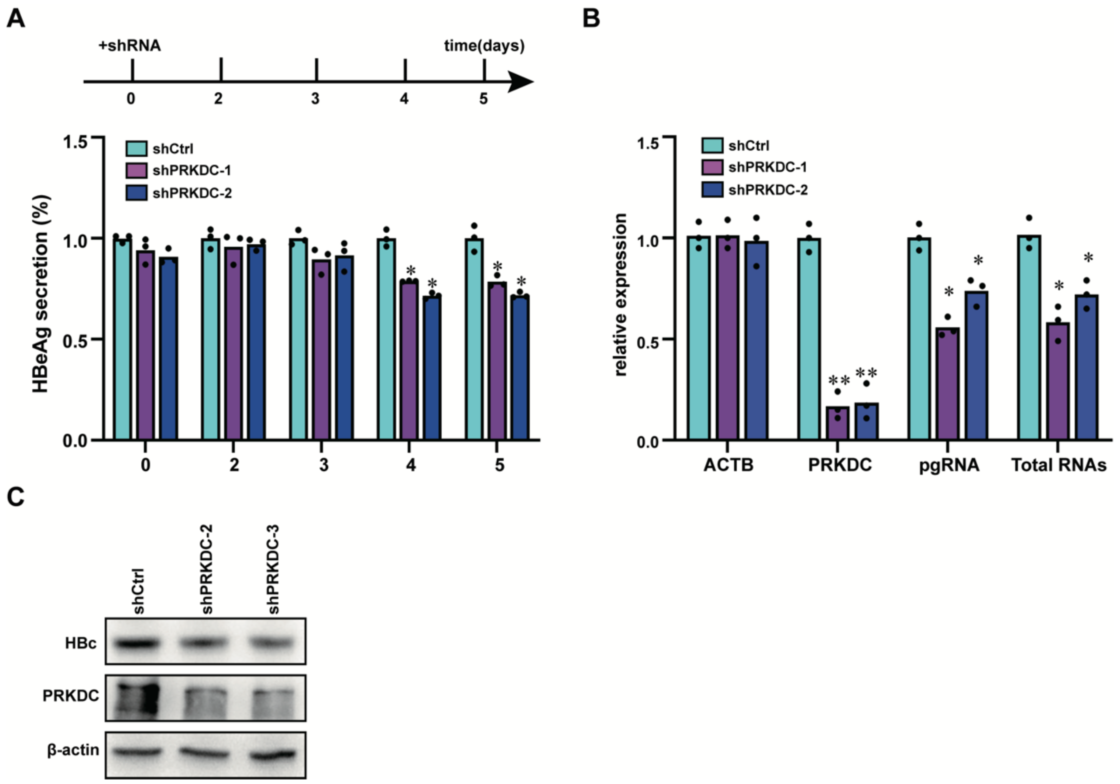

Figure S6. PRKDC knockdown in HepG2.2.15 inhibited HBeAg secretion and HBV transcription. (A) (Upper) The timeline of shRNA knockdown in HepG2.2.15. HepG2.2.15 cells were seeded in wells, and the PRKDC shRNAs virus were added at the same time. Two days later, the supernatants and cells were collected for detection of HBV HBeAg, pgRNA and total HBV RNAs by Elisa or RT-qPCR. Subsequently, the cells were collected for the same detections each day until the indicated time. WB for HBc antibody was performed at last day. (Bottom) PRKDC suppression decreased HBeAg secretion as detected by ELISA kit. (B) HBV RNAs, pgRNA, and PRKDC were analyzed by real-time PCR using specific primers. (C) Western blot analysis with specific antibodies against HBc and PRKDC. β-actin as loading control.

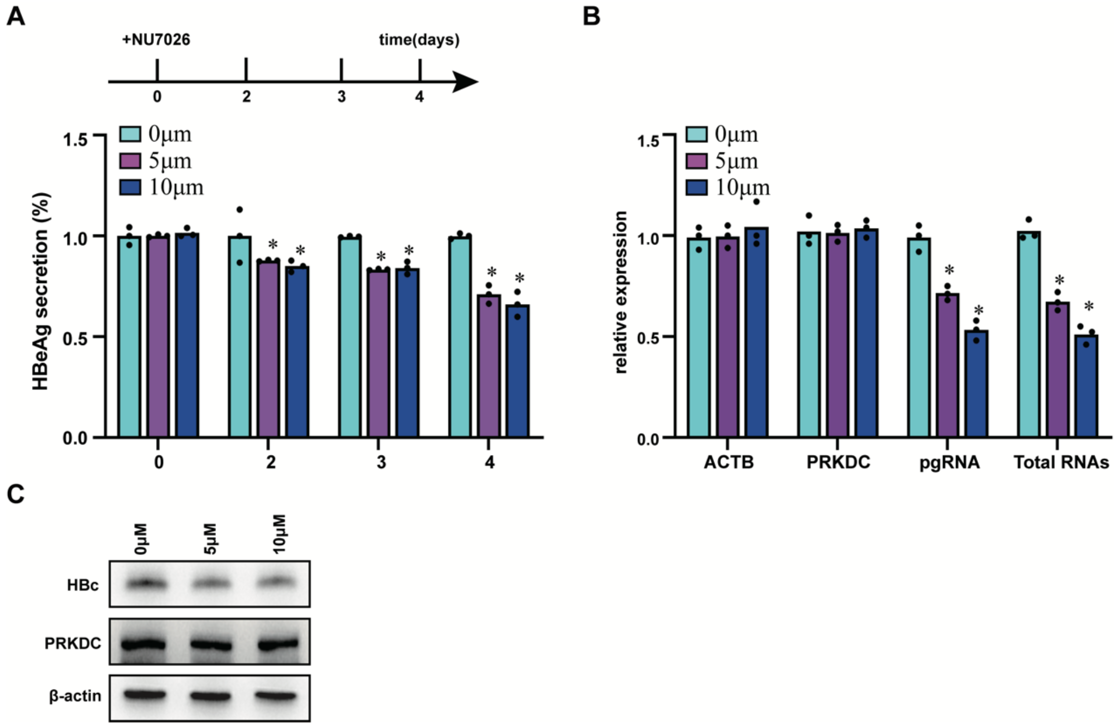

Figure S7 NU7026 treatment in HepG2.2.15 inhibited HBeAg secretion and HBV transcription. (A) (Upper) The timeline of NU7026 treating in HepG2.2.15. HepG2.2.15 cells were seeded in wells, and the NU7026 was added at the same time. Two days later, the supernatants and cells were collected for detection of HBV HBeAg, pgRNA and total HBV RNAs by Elisa or RT-qPCR. Subsequently, the cells were collected for the same detections each day until the indicated time. WB for HBc antibody was performed at last day. (Bottom) NU7026 treatment decreased HBeAg secretion as detected by ELISA kit. (B) HBV RNAs, pgRNA, and PRKDC were analyzed by real-time PCR using specific primers. (C) Western blot analysis with specific antibodies against HBc and PRKDC. β-actin as loading control.

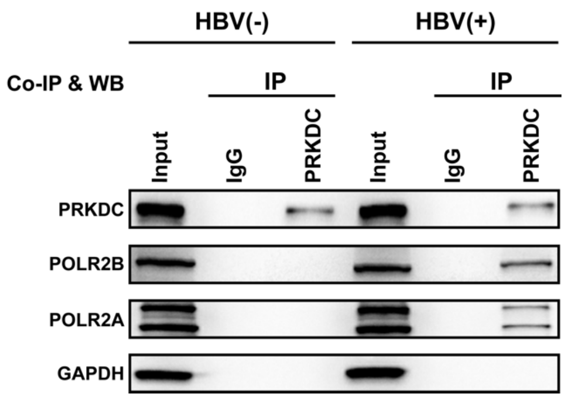

Figure S8 PRKDC interacts with POLR2A and POLR2B. Endogenous co-immunoprecipitation with PRKDC was carried out in HBV-infected HepG2-NTCP and mock-infected HepG2-NTCP cells using indicated antibodies and blotted with specific antibodies.

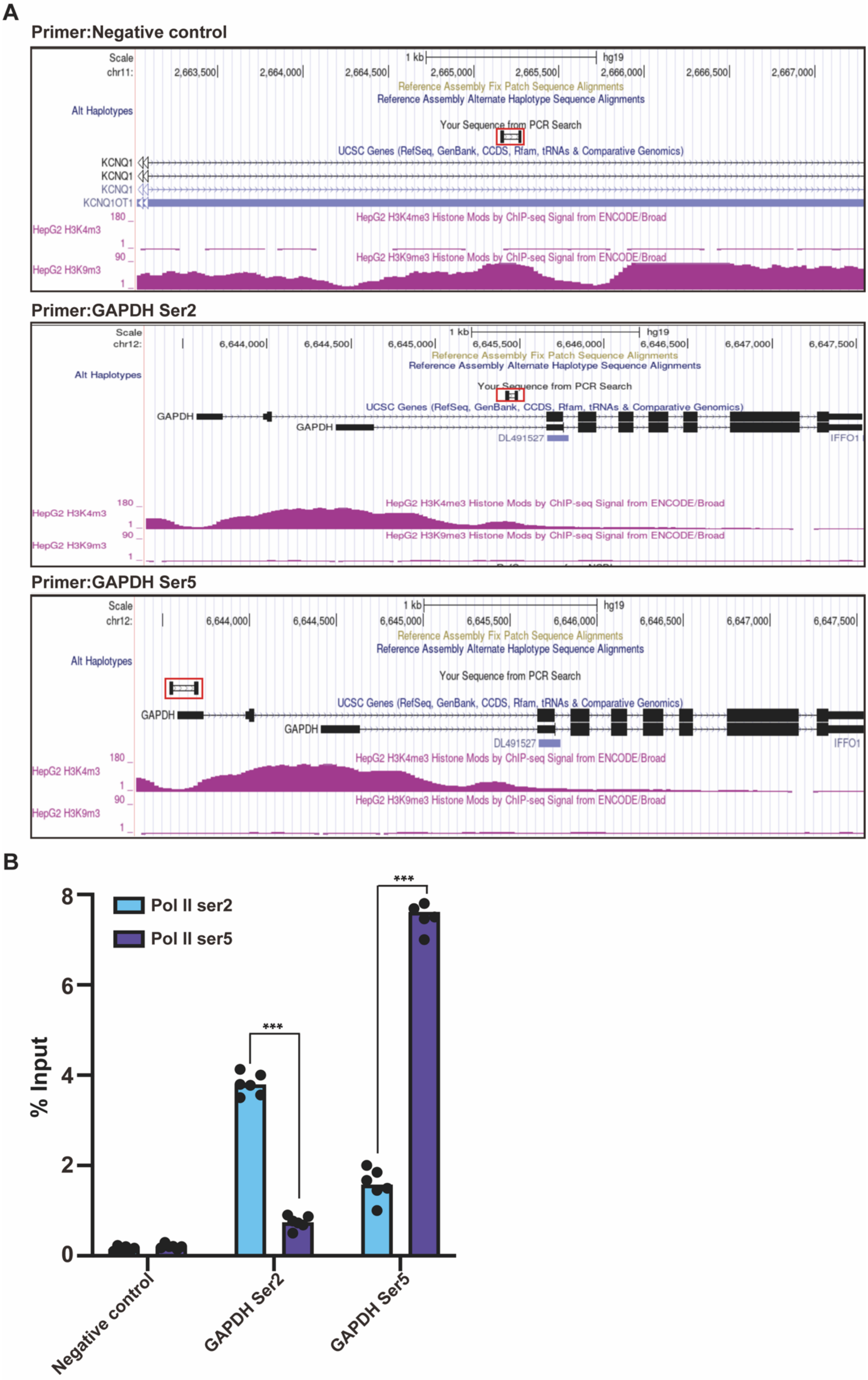

Figure S9. The antibody specificity of Pol II Ser2, and Pol II Ser5 were detected

by ChIP-qPCR. (A) The designed primers for Negative control (Upper), GAPDH Ser2 (Middle), and GAPDH Ser5 (Bottom) were analyzed in UCSC genome website. (B) HepG2-NTCP cells were cross-linked by 4% formaldehyde solution, sonicated, and immunoprecipitated with indicated antibodies. The enrichment was expressed as % input using above designed primers. ***$P < 0.001$.

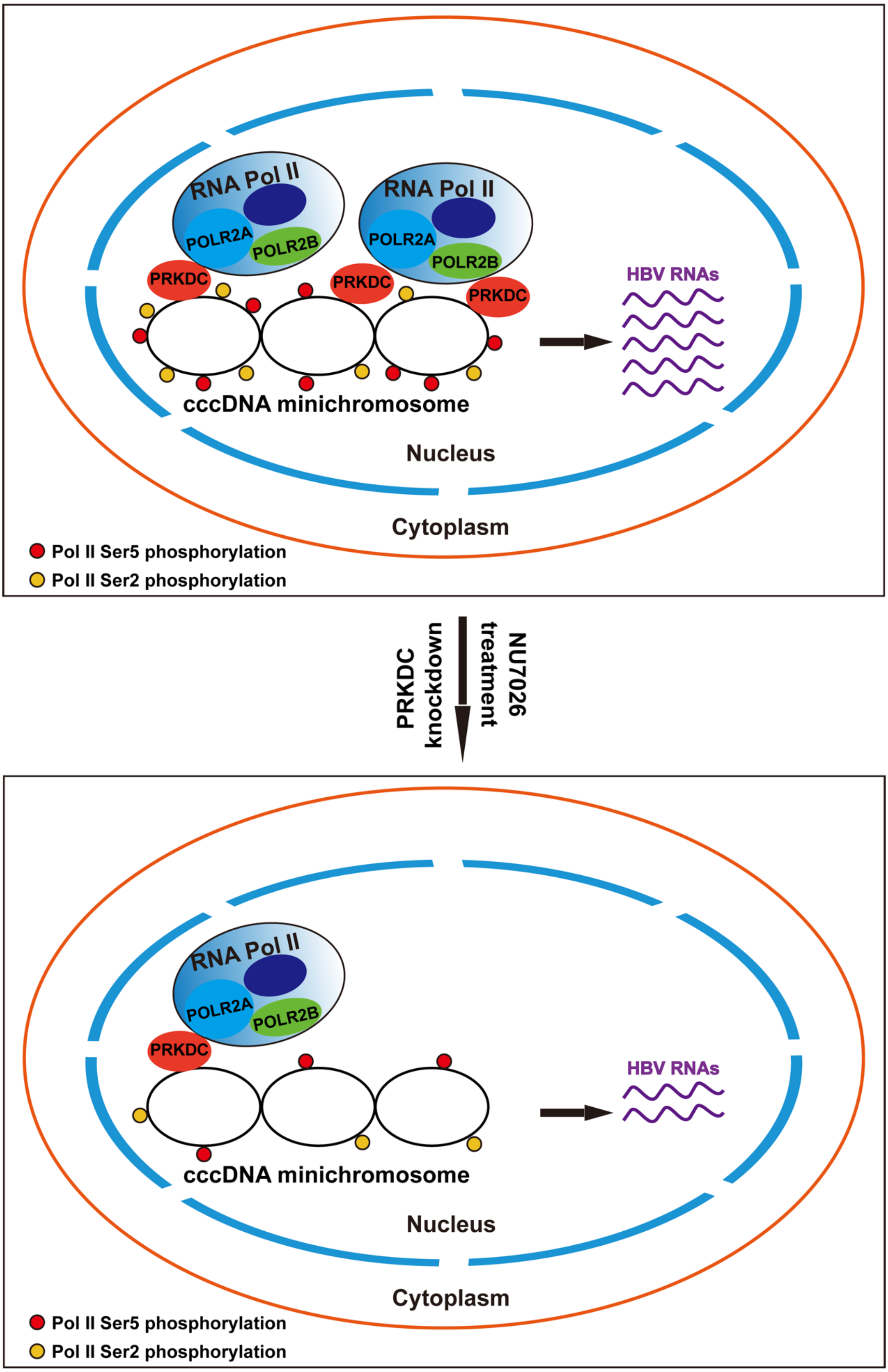

Figure S10. A proposed model for PRKDC-mediated recruitment and

phosphorylation of Pol II among HBV cccDNA minichromosome. In the nucleus, PRKDC facilitates HBV cccDNA transcription through interacting with POLR2A and PLOR2B, the largest and second largest subunit of RNA Pol II, the recruitment of Pol II to cccDNA depends on its kinase function. PRKDC knockdown or NU7026 treatment decrease the enrichment of Pol II associated with cccDNA minichromosome, and Ser5 or Ser2 phosphorylation levels are inhibited, leading to the decrease of HBV cccDNA transcription.

Supplementary Table S1 Proteins identified by MS

Accession	Gene names	sgGal4	8sgRNAs
		Protein score	Protein score
O43670	ZNF207	0.00	67.32
Q96KR1	ZFR	0.00	70.55
Q9H5U6	ZCCHC4	0.00	35.98
P27348	YWHAQ	89.41	78.55
P12956	XRCC6	40.57	86.66
P13010	XRCC5	127.68	131.86
Q9HAV4	XPO5	31.95	40.71
O14980	XPO1	0.00	78.97
Q9NP79	VTA1	0.00	54.42
Q9Y277	VDAC3	103.30	156.06
P45880	VDAC2	61.07	57.08
P21796	VDAC1	104.96	175.92
P55072	VCP	81.42	138.06
P26640	VARS1	41.12	45.29
O95292	VAPB	0.00	64.13
Q93009	USP7	72.07	123.51
P45974	USP5	0.00	55.98
P51784	USP11	0.00	60.48
O60763	USO1	0.00	29.57
Q92900	UPF1	94.25	51.65
O60701	UGDH	45.34	100.42
O94874	UFL1	0.00	51.39
P0CG48	UBC	56.96	42.38
P22314	UBA1	0.00	71.85
Q16881	TXNRD1	103.20	115.27
P49411	TUFM	353.20	223.62
P68371	TUBB4B	997.59	928.86
Q13509	TUBB3	0.00	692.47
Q13885	TUBB2A	828.97	868.28
P07437	TUBB	956.27	830.47
A6NHL2	TUBAL3	170.61	332.20
Q9BQE3	TUBA1C	728.48	743.15
Q13263	TRIM28	153.59	322.32
Q14258	TRIM25	0.00	45.70
P12270	TPR	44.22	70.80
O14773	TPP1	47.61	41.21
Q3LXA3	TKFC	73.05	0.00
Q3ZCQ8	TIMM50	0.00	44.76
Q9NXG2	THUMPD1	0.00	67.88
P17987	TCP1	52.45	72.75

Q13148	TARDBP	127.29	133.12
Q92804	TAF15	0.00	110.35
Q16563	SYPL1	0.00	55.54
P0DMM9	SULT1A3	40.34	58.97
Q13586	STIM1	0.00	48.60
Q9BXP5	SRRT	0.00	51.00
Q15005	SPCS2	0.00	42.74
P14678	SNRPB	26.13	48.24
A6NHR9	SMCHD1	40.03	67.87
P08195	SLC3A2	39.75	0.00
P11166	SLC2A1	49.54	69.79
P12236	SLC25A6	0.00	115.56
P05141	SLC25A5	113.43	0.00
Q00325	SLC25A3	47.10	0.00
Q9UJS0	SLC25A13	123.04	345.77
P53007	SLC25A1	50.71	85.90
Q9H9B4	SFXN1	52.16	122.96
P23246	SFPQ	111.91	145.27
Q15637	SF1	0.00	41.51
P01008	SERPINC1	0.00	55.85
P30740	SERPINB1	0.00	53.06
Q9UHD8	SEPTIN9	36.48	89.48
O94979	SEC31A	132.58	209.36
P53992	SEC24C	0.00	71.54
O95486	SEC24A	0.00	81.11
Q15436	SEC23A	57.69	100.18
Q15020	SART3	0.00	41.70
Q9Y3Z3	SAMHD1	60.81	112.25
Q9P2E9	RRBP1	69.84	89.35
P08865	RPSA	72.73	49.98
P04843	RPN1	0.00	108.94
P05388	RPLP0	152.54	104.26
Q6NUM9	RETSAT	0.00	62.11
P46063	RECQL	0.00	35.84
Q96E39	RBMXL1	0.00	81.94
Q96PK6	RBM14	0.00	73.08
Q9NTZ6	RBM12	0.00	42.12
P49792	RANBP2	55.30	141.24
P62826	RAN	0.00	46.81
Q92878	RAD50	0.00	77.57
P51148	RAB5C	0.00	57.55
P47897	QARS1	48.60	50.16
P32322	PYCR1	36.77	0.00

P26599	PTBP1	71.33	86.46
Q06323	PSME1	40.49	92.81
Q13200	PSMD2	0.00	52.75
P62333	PSMC6	88.04	74.07
P35998	PSMC2	0.00	113.17
P62191	PSMC1	41.69	45.48
O14744	PRMT5	61.69	61.76
Q99873	PRMT1	0.00	90.25
P78527	PRKDC	0.00	821.04
P30041	PRDX6	0.00	63.85
P30048	PRDX3	76.52	0.00
P32119	PRDX2	49.54	61.03
Q06830	PRDX1	65.65	42.94
O15355	PPM1G	45.27	54.89
P16435	POR	0.00	92.79
P30876	POLR2B	8.45	50.98
Q15149	PLEC	0.00	111.56
P14618	PKM	37.60	51.62
O43175	PHGDH	96.98	0.00
P35232	PHB	189.33	129.78
P08237	PFKM	42.19	0.00
P17858	PFKL	0.00	63.12
P12955	PEPD	54.65	54.07
Q15084	PDIA6	54.34	209.10
P11177	PDHB	74.03	68.33
Q53EL6	PDCD4	0.00	39.70
Q99447	PCYT2	0.00	43.42
P49585	PCYT1A	0.00	55.79
P05166	PCCB	159.25	141.91
P05165	PCCA	2017.85	2062.46
Q15365	PCBP1	145.45	194.25
P11498	PC	3764.27	3336.84
P09874	PARP1	38.19	67.71
Q99497	PARK7	37.35	0.00
O43252	PAPSS1	0.00	44.11
P07237	P4HB	130.88	62.28
Q9BZF1	OSBPL8	0.00	64.25
P52948	NUP98	0.00	44.19
Q92621	NUP205	0.00	65.86
Q12769	NUP160	0.00	95.01
Q8WUM0	NUP133	0.00	66.24
P57740	NUP107	0.00	57.77
P46459	NSF	37.71	0.00

P06748	NPM1	80.87	125.80
Q15233	NONO	62.67	0.00
P35579	MYH9	95.73	240.53
P35580	MYH10	69.09	165.90
Q9Y6C9	MTCH2	42.78	41.55
Q13724	MOGS	0.00	44.23
Q9NP71	MLXIPL	0.00	54.50
Q9ULK4	MED23	0.00	42.56
Q96RQ3	MCCC1	869.84	949.04
Q7Z434	MAVS	0.00	65.96
P43243	MATR3	0.00	212.47
P56192	MARS1	70.14	55.99
Q96AG4	LRRC59	48.52	49.75
P42704	LRPPRC	0.00	65.31
P20700	LMNB1	80.86	78.87
P02545	LMNA	555.85	713.49
P49257	LMAN1	63.28	91.14
P17931	LGALS3	0.00	38.65
P00338	LDHA	0.00	56.33
Q9Y4W2	LAS1L	0.00	60.22
P13473	LAMP2	0.00	55.68
P35527	KRT9	593.38	855.79
P05787	KRT8	238.06	115.65
P08727	KRT19	127.71	0.00
P05783	KRT18	71.85	0.00
P08779	KRT16	117.97	274.34
P13645	KRT10	510.39	498.32
P04264	KRT1	900.49	1216.98
Q14974	KPNB1	0.00	56.32
Q92945	KHSRP	131.18	451.54
Q5VWX1	KHDRBS2	53.96	0.00
Q07666	KHDRBS1	0.00	75.48
P14923	JUP	37.98	0.00
P46940	IQGAP1	0.00	43.10
Q13418	ILK	0.00	52.02
Q12906	ILF3	110.39	340.22
Q12905	ILF2	66.62	108.73
P01834	IGKC	87.34	0.00
P11717	IGF2R	0.00	47.40
O00425	IGF2BP3	0.00	41.52
Q9NZI8	IGF2BP1	0.00	38.33
Q7Z6Z7	HUWE1	0.00	47.07
O43719	HTATSF1	0.00	43.11

P10809	HSPD1	256.45	87.26
P38646	HSPA9	197.37	317.74
P11142	HSPA8	320.42	464.52
P11021	HSPA5	242.57	238.09
P0DMV9	HSPA1B	156.76	206.89
P14625	HSP90B1	0.00	72.17
P08238	HSP90AB1	495.26	765.67
P07900	HSP90AA1	324.79	536.08
P51659	HSD17B4	52.43	57.01
Q86YZ3	HRNR	61.73	118.24
Q1KMD3	HNRNPUL2	0.00	104.26
Q9BUJ2	HNRNPUL1	0.00	69.52
Q00839	HNRNPU	256.67	386.76
O43390	HNRNPR	0.00	127.25
P52272	HNRNPM	317.68	371.61
P14866	HNRNPL	62.37	411.44
P61978	HNRNPK	448.97	695.08
P31943	HNRNPH1	134.63	348.22
P52597	HNRNPF	118.02	175.40
O14979	HNRNPDL	82.97	124.87
B2RXH8	HNRNPCL2	0.00	64.78
P51991	HNRNPA3	174.35	335.71
P22626	HNRNPA2B1	265.37	506.12
Q32P51	HNRNPA1L2	157.10	227.01
Q2TB90	HKDC1	0.00	205.28
O94992	HEXIM1	0.00	41.05
P51858	HDGF	45.98	75.36
P02042	HBD	62.09	44.78
P55084	HADHB	0.00	54.66
P40939	HADHA	43.13	72.27
P16403	H1-2	82.07	101.45
Q9Y5Q9	GTF3C3	0.00	49.82
P78417	GSTO1	0.00	56.07
P00390	GSR	0.00	41.14
P49915	GMPS	0.00	39.46
O76003	GLRX3	0.00	57.90
Q92616	GCN1	0.00	43.57
P51570	GALK1	62.05	74.08
P11413	G6PD	46.91	47.30
P35637	FUS	91.59	44.75
Q96AE4	FUBP1	0.00	339.25
P02751	FN1	111.96	189.18
O75369	FLNB	406.17	657.03

P21333	FLNA	229.60	335.01
Q13045	FLII	0.00	49.56
Q5D862	FLG2	52.47	53.26
P20930	FLG	42.12	0.00
P02679	FGG	103.21	235.63
P02675	FGB	76.30	213.55
P02671	FGA	176.00	448.49
P37268	FDFT1	47.69	109.26
P09467	FBP1	151.24	90.11
P49327	FASN	844.66	1484.73
Q9Y4F1	FARP1	0.00	132.19
Q52LJ0	FAM98B	0.00	92.80
Q9Y6X4	FAM169A	176.33	113.71
Q01844	EWSR1	0.00	190.52
O95571	ETHE1	37.02	0.00
P38117	ETFB	0.00	36.16
Q9BSJ8	ESYT1	56.64	74.38
O94905	ERLIN2	66.17	51.74
P07814	EPRS1	0.00	115.99
Q9HC35	EML4	78.39	187.59
Q15717	ELAVL1	0.00	37.06
Q99613	EIF3C	0.00	41.30
P55884	EIF3B	49.19	71.47
Q14152	EIF3A	0.00	46.55
Q15029	EFTUD2	0.00	42.13
P13639	EEF2	80.43	62.18
P26641	EEF1G	59.54	68.51
P68104	EEF1A1	246.62	216.29
Q13409	DYNC1I2	38.86	0.00
Q14204	DYNC1H1	42.38	422.83
Q99956	DUSP9	47.41	71.78
P15924	DSP	0.00	58.43
Q02413	DSG1	0.00	46.05
Q16555	DPYSL2	37.87	0.00
P31689	DNAJA1	37.03	0.00
P36957	DLST	0.00	36.47
P10515	DLAT	43.58	46.55
Q9Y2L1	DIS3	0.00	74.21
Q08211	DHX9	40.65	242.30
Q92620	DHX38	0.00	66.44
O43143	DHX15	0.00	156.51
Q13268	DHRS2	132.85	175.47
Q9UBM7	DHCR7	0.00	52.42

Q15392	DHCR24	0.00	57.47
P17844	DDX5	0.00	216.42
Q7L014	DDX46	0.00	44.64
Q86XP3	DDX42	0.00	67.45
O00571	DDX3X	71.10	158.16
O00148	DDX39A	0.00	71.05
Q92841	DDX17	138.20	465.07
Q5TDH0	DDI2	44.13	0.00
P81605	DCD	0.00	52.56
O60716	CTNND1	0.00	54.74
P01040	CSTA	45.59	61.78
Q9BRF8	CPPED1	0.00	75.80
Q9UBF2	COPG2	51.32	45.96
P53618	COPB1	0.00	75.68
P53621	COPA	81.61	325.31
Q13057	COASY	0.00	34.01
Q00610	CLTC	239.42	335.84
P09496	CLTA	0.00	40.10
O00299	CLIC1	73.29	0.00
P23528	CFL1	42.92	0.00
Q16543	CDC37	45.56	73.49
Q8N163	CCAR2	0.00	83.06
Q01518	CAP1	0.00	42.69
Q86VP6	CAND1	0.00	87.98
Q9NZT1	CALML5	63.36	0.00
P27708	CAD	0.00	54.86
P48047	ATP5PO	38.67	0.00
O75947	ATP5PD	70.41	0.00
P36542	ATP5F1C	41.14	54.94
P25705	ATP5F1A	361.77	316.52
P05023	ATP1A1	0.00	152.30
P40616	ARL1	0.00	38.12
P18085	ARF4	51.00	78.92
P07741	APRT	50.41	50.20
P04114	APOB	0.00	60.35
O14617	AP3D1	0.00	53.42
P63010	AP2B1	0.00	68.53
O95782	AP2A1	0.00	55.78
O43747	AP1G1	0.00	81.59
P20073	ANXA7	0.00	45.80
P08133	ANXA6	0.00	43.03
P04075	ALDOA	85.73	93.61
P54886	ALDH18A1	38.69	52.63

P02768	ALB	64.83	103.30
P42330	AKR1C3	62.11	50.72
O43823	AKAP8	0.00	41.41
P27144	AK4	36.15	0.00
Q13155	AIMP2	0.00	64.55
P23526	AHCY	0.00	62.76
Q96SZ5	ADO	40.67	40.46
O43707	ACTN4	0.00	120.81
P12814	ACTN1	53.07	0.00
P63267	ACTG2	245.88	298.42
Q9ULC5	ACSL5	157.69	262.56
O60488	ACSL4	0.00	79.19
Q96CM8	ACSF2	61.51	0.00
P53396	ACLY	118.81	412.26
P49748	ACADVL	70.40	0.00
Q13085	ACACA	533.76	867.63

Supplementary Table S2 The sequences of the primers used in this study

Gene name		Primer Sequence (5'→3')
**Primers for detection of expression**		
ACTB	F	ACTCTTCCAGCCTTCCTTCC
	R	TGTTGGCGTACAGGTCTTTG
PRKDC	F	CATGGAAGAAGATCCCCAGA
	R	TGGGCACACCACTTTAACAA
HBV 3.5-kb pgRNA	F	GCCTTAGAGTCTCCTGAGCA
	R	GAGGGAGTTCTTCTTCTAGG
Total HBV RNAs	F	ACCGACCTTGAGGCATACTT
	R	GCCTACAGCCTCCTAGTACA
HBV HBC	F	TTCGCACTCCTCCAGCTTAT
	R	GGCGAGGGAGTTCTTCTTCT
**Primers for quantification of viral titer**		
HBV DNA	F	CCTAGTAGTCAGTTATGTCAAC
	R	TCTATAAGCTGGAGGAGTGCGA
**Primers for ChIP-qPCR**		
HBV cccDNA	F	GTGCACTTCGCTTCACCTCT
	R	AGCTTGGAGGCTTGAACAGT
HBV cccDNA probe		5′-FAM-ACGTCGCATGGAGACCACCGTGAACGCC-TAMRA-3′
MYH7	F	AGAAGCTGCGCTCAGACCTGTCTCG
	R	TCCAGGTCCCGCCGCATCTT
GAPDH	F	TACTAGCGGTTTTACGGGCG
	R	TCGAACAGGAGGAGCAGAGAGCGA
GAPDH-Pol II Ser2	F	CTCCTGGAAGGGCTTCGTAT
	R	CTTAAGGCATGGCTGCAACT
GAPDH-Pol II Ser5	F	TACTAGCGGTTTTACGGGCG
	R	TCGAACAGGAGGAGCAGAGAGCGA
**Primers for PRKDC knockdown target sequences**		
shPRKDC-1	F	GCCTTACTAGAAGCTATATTG
shPRKDC-2	F	CCTGAAGTCTTTACAACATAT
**Primers for sgRNAs**		
sgRNA-1	F	CACCGTCTAGAAGATCTCGTACTGA
	R	AAACTCAGTACGAGATCTTCTAGAC
sgRNA-2	F	CACCGACTACTGTTGTTAGACGACG
	R	AAACCGTCGTCTAACAACAGTAGTC
sgRNA-3	F	CACCGGATTGAGATCTTCTGCGACG
	R	AAACCGTCGCAGAAGATCTCAATCC
sgRNA-4	F	CACCGATTTGGTGTCTTTCGGAGTG
	R	AAACCACTCCGAAAGACACCAAATC
sgRNA-5	F	CACCGCAGTTATGTCAACACTAATA
	R	AAACTATTAGTGTTGACATAACTGC
sgRNA-6	F	CACCGAGGAGGCTGTAGGCATAAAT

	R	AAACATTTATGCCTACAGCCTCCTC
sgRNA-7	F	CACCGAGCTTGGAGGCTTGAACAGT
	R	AAACACTGTTCAAGCCTCCAAGCTC
sgRNA-8	F	CACCGCAAGCCTCCAAGCTGTGCCT
	R	AAACAGGCACAGCTTGGAGGCTTGC
sgGal4	F	CACCGAACGACTAGTTAGGCGTGTA
	R	AAACTACACGCCTAACTAGTCGTTC
