# Supplementary material for: PRKDC promotes hepatitis B virus transcription through enhancing the binding of RNA Pol II to cccDNA
Source: Cell Death Dis. 2022 Apr 25;13(4):404. doi: 10.1038/s41419-022-04852-3 (PMC9038722; doi:10.1038/s41419-022-04852-3)

Figure 1B

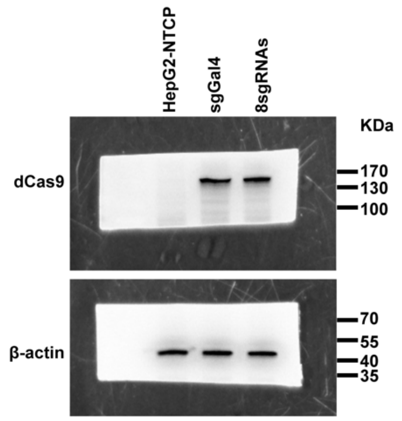

Figure 1E

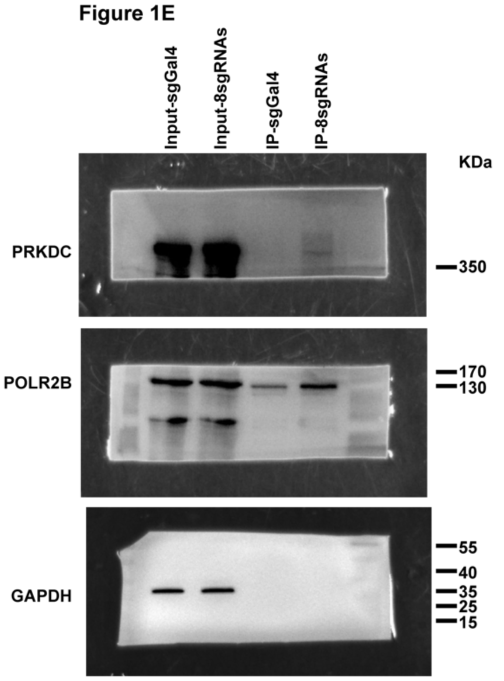

Figure 1D

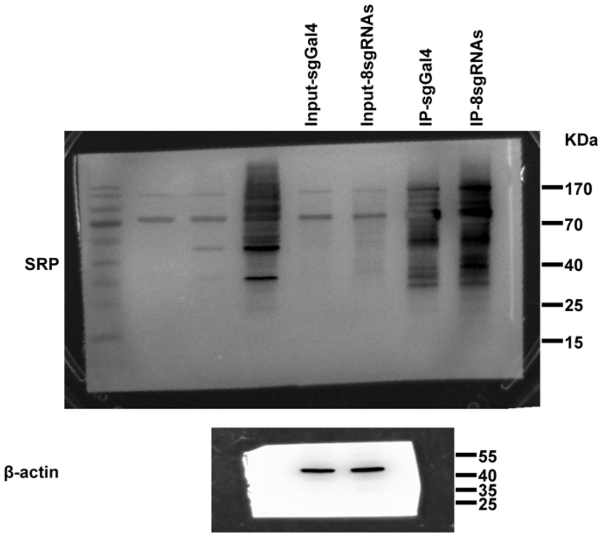

Figure 2A

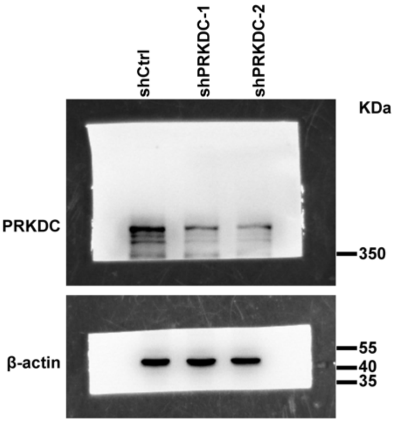

Figure 2F

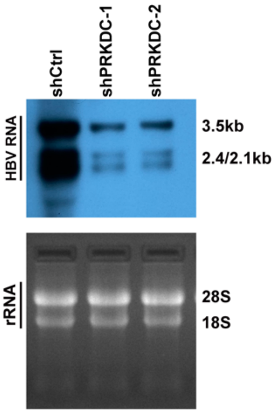

Figure 2G

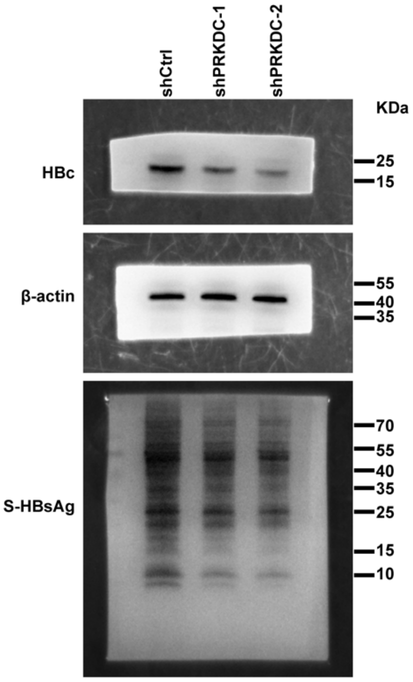

Figure 3E

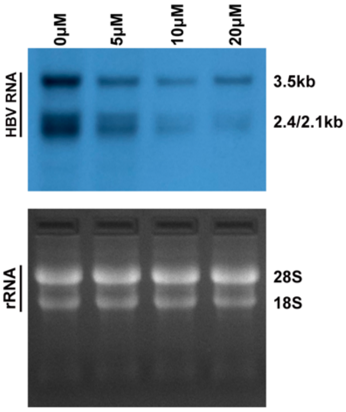

Figure 3F

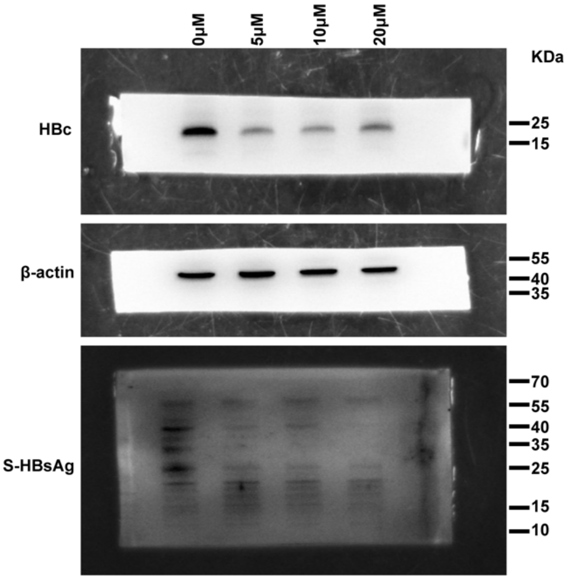

Figure 5C upper-left

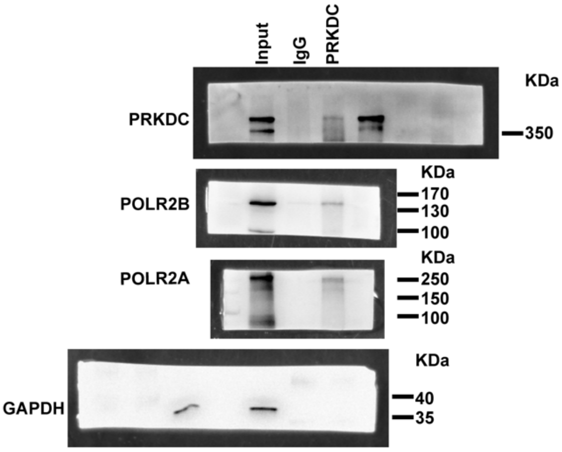

Figure 5C upper-middle

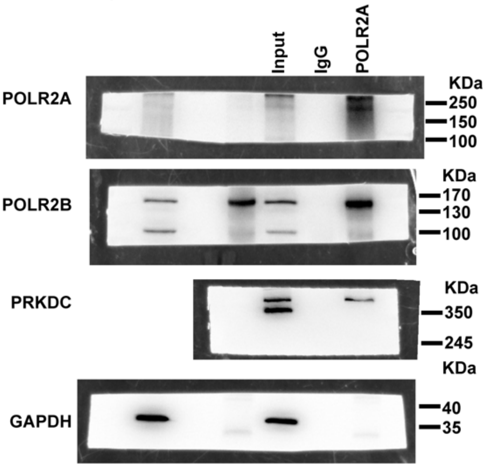

Figure 5C upper-right

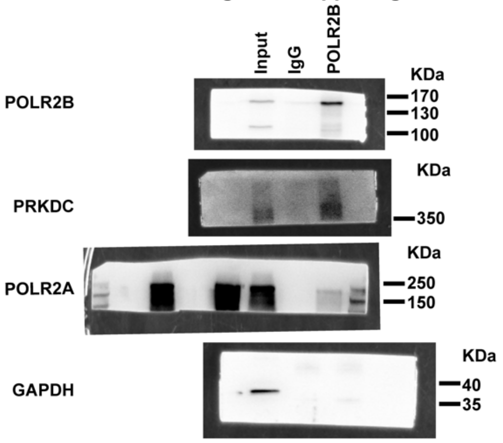

Figure 5C bottom-left

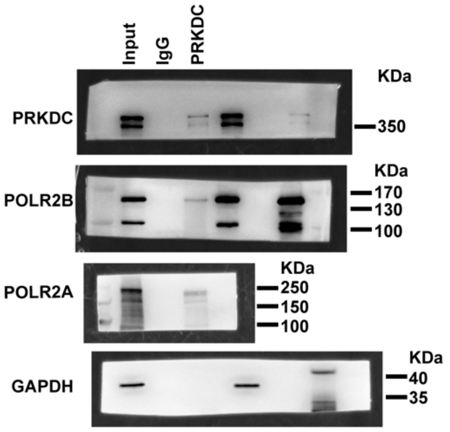

Figure 5C bottom-middle

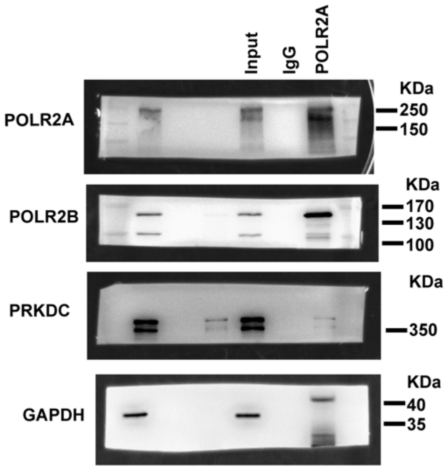

Figure 5C bottom-right

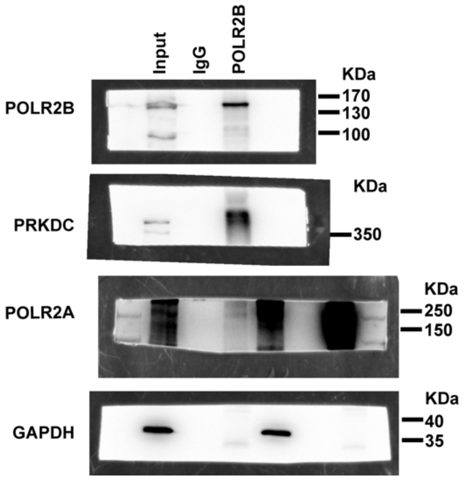

Figure 7A

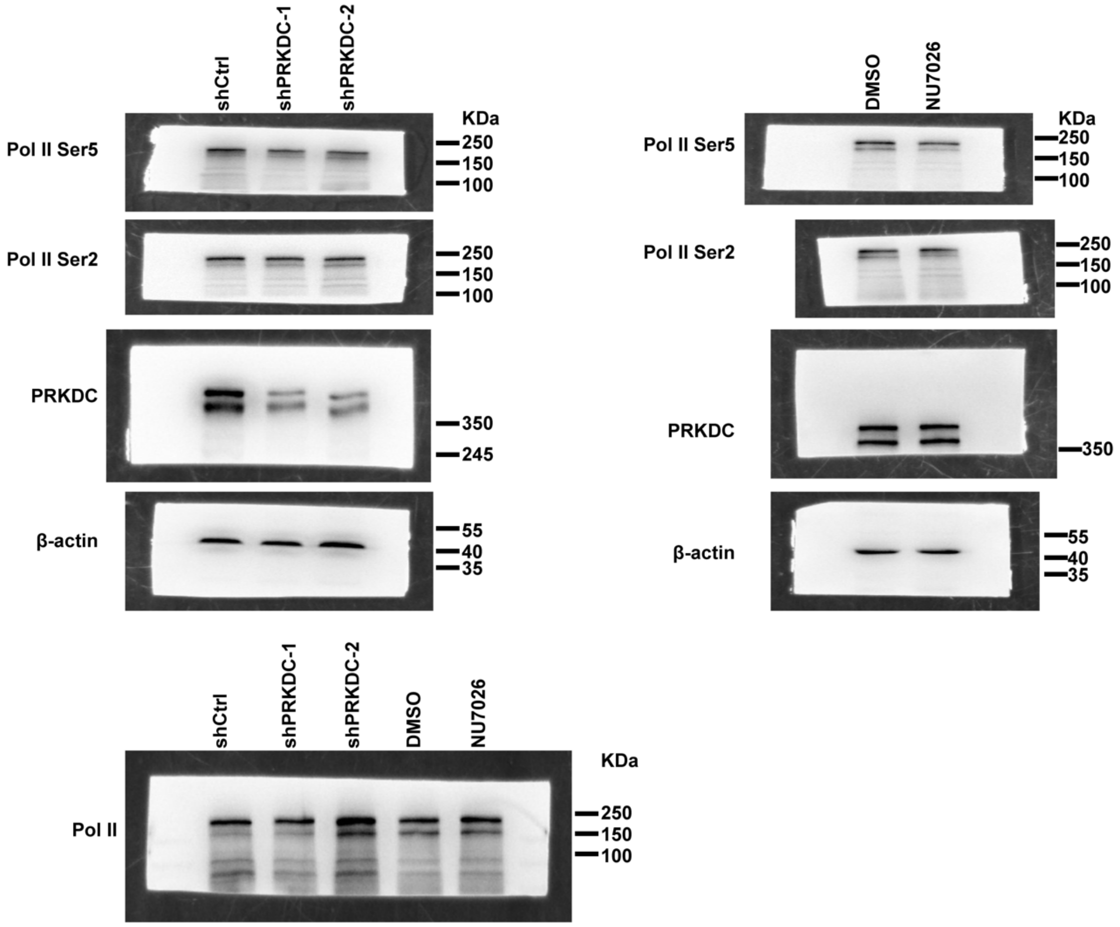
shCtrl
shPRKDC-1
shPRKDC-2
KDa
Pol II Ser5
250
150
100
Pol II Ser2
250
150
100
PRKDC
350
245
β-actin
55
40
35
DMSO
NU7026
KDa
Pol II Ser5
250
150
100
Pol II Ser2
250
150
100
PRKDC
350
β-actin
55
40
35
shCtrl
shPRKDC-1
shPRKDC-2
DMSO
NU7026
KDa
Pol II
250
150
100

Supplymentary Figure 4A

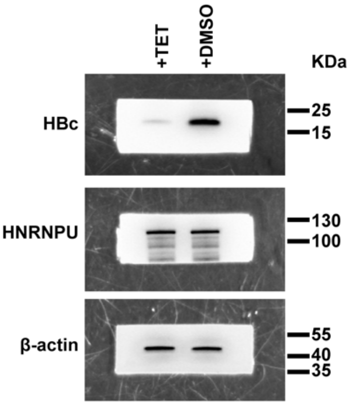

Supplymentary Figure 4B

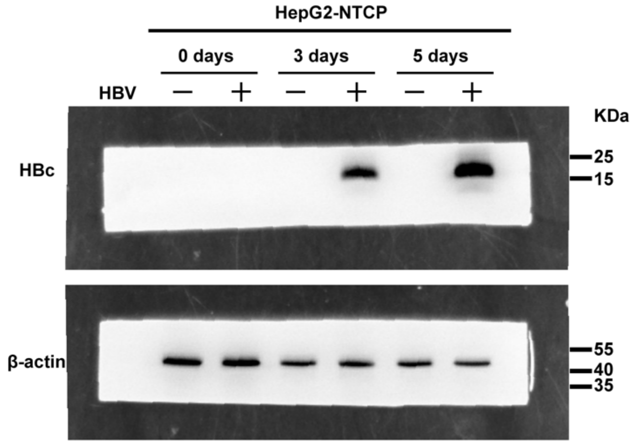

Supplymentary Figure 6C

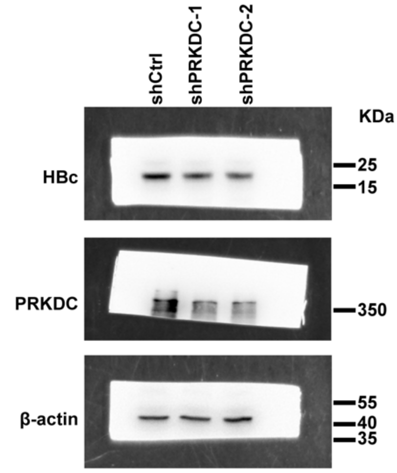

Supplymentary Figure 7C

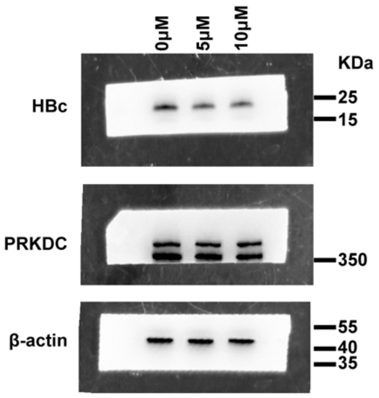

Supplymentary Figure 8

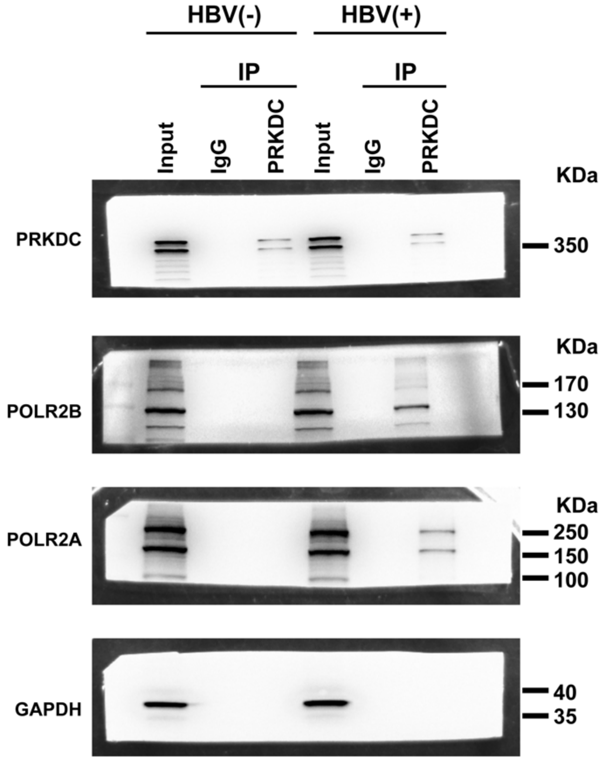

Supplement: Supplementary file 3 — western blot raw data [file 41419_2022_4852_MOESM3_ESM.pdf]
